# Nutritional Composition, Health Benefits, and Application Value of Edible Insects: A Review

**DOI:** 10.3390/foods11243961

**Published:** 2022-12-07

**Authors:** Yaxi Zhou, Diandian Wang, Shiqi Zhou, Hao Duan, Jinhong Guo, Wenjie Yan

**Affiliations:** 1 College of Biochemical Engineering, Beijing Union University, No.18, Chaoyang District 3, Futou, Beijing 100023, China; 2 Beijing Key Laboratory of Bioactive Substances and Functional Food, College of Biochemical Engineering, Beijing Union University, 197 North Tucheng West Road, Beijing 100023, China

**Keywords:** edible insects, active ingredients, nutrition, pharmacological functions, health benefits, safety, applications

## Abstract

For thousands of years, edible insects have been used as food to alleviate hunger and improve malnutrition. Some insects have also been used as medicines because of their therapeutic properties. This is not only due to the high nutritional value of edible insects, but more importantly, the active substances from edible insects have a variety of biofunctional activities. In this paper, we described and summarized the nutritional composition of edible insects and discussed the biological functions of edible insects and their potential benefits for human health. A summary analysis of the findings for each active function confirms that edible insects have the potential to develop functional foods and medicines that are beneficial to humans. In addition, we analyzed the issues that need to be considered in the application of edible insects and the current status of edible insects in food and pharmaceutical applications. We concluded with a discussion of regulations related to edible insects and an outlook on future research and applications of edible insects. By analyzing the current state of research on edible insects, we aim to raise awareness of the use of edible insects to improve human health and thus promote their better use and development.

## 1. Introduction

The beginning of human consumption of insects dates back 7000 years [1]. More than 2300 species of edible insects exist worldwide, primarily in Africa, Asia, and the Americas, according to statistics [2]. Beetles, caterpillars, bees, wasps, ants, grasshoppers, locusts, crickets, real bugs, dragonflies, termites, flies, cockroaches, and other orders are the most popular food insects [3,4,5]. The most consumed insect species globally are those of the orders Isoptera, Lepidoptera, Orthoptera, and Hymenoptera [6]. These edible insects live largely on land, although some can also be found in the biotic environment. Natural sources of edible insects have traditionally been used. In recent years, several insect species, such as yellow mealworms, locusts, and crickets, have been farmed on a huge scale [7].

Food that is both healthy and sustainable is becoming increasingly scarce in modern society. As a result, finding new food alternatives to traditional food sources is critical for humanity’s long-term development. Insects could be a nice option. Numerous studies have shown that proteins, lipids, and other elements from edible insects can be used to replace traditional sources of nutrition [8]. Insects, when consumed as food or feed, reduce human demand for animal protein and, as a result, natural resource usage [9]. At any stage of growth, edible insects can be used as a highly nutritious food source since they are packed with nutrients. Because of their high protein and fat content, consumers are particularly fond of immature insects like larvae and pupae [10]. In comparison to other food sources, edible insects are not only nutritious, but also delicious, healthy, environmentally beneficial, and sustainable [11].

In addition to being used as food, edible insects are also utilized in pharmaceuticals to cure human illnesses [12,13]. Numerous amino acids, peptides, functional lipids, minerals, vitamins, fiber, and secondary metabolites are among the functional active compounds found in insects. The majority of these active ingredients are found in insects’ bodies, eggs, and secretions [14]. Certain edible insects have long been used as medications to maintain human health because they contain these useful compounds. Functional ingredients from edible insects have been demonstrated in numerous in vivo and in vitro studies to possess gastrointestinal protection, antioxidant and anti-inflammatory activity, antibacterial activity, immunomodulatory effects, blood glucose and lipid regulation, hypotensive effects, and a decreased risk of cardiovascular disease [15,16].

Even though edible insects are incredibly nutritious and healthy, they are not widely accepted or used. The good news is that the edible insect sector has gained a lot of attention from firms and governments in recent years, and edible insect rules are being created and passed all around the world [17]. The downside is that most laws concentrate on the nutrition and safety of edible insects as food, rather than on the functional compounds found in edible insects [18]. Scientific studies have also concentrated on nutritional composition, insect processing, and food development, while the exploration and application of edible insects’ biological functions need to be further explored and studied [19].

We described and summarized the nutritional composition of edible insects in this paper, emphasizing their feasibility and necessity as a source of high nutrient content. Following that, we concentrated on the biological activities of edible insects and their potential advantages to human health. By aggregating the results of the investigations for each of the active functions, it was established that edible insects have the potential to generate functional food and pharmaceutical items that are helpful to humans. Furthermore, we investigated many challenges that must be addressed in the use of edible insects, as well as the current state of edible insects in food and pharmaceutical applications. We closed with a discussion of edible insect restrictions and suggested many topics to be considered in future edible insect research and implementation. The purpose of this study is to investigate the therapeutic potential of edible insects by reviewing existing research on edible insects; raising concerns about the use of edible insects to enhance human health and promoting the better use and development of edible insects.

## 2. Nutritional Value of Insects

The nutritional value of insects is extremely high, and the main nutrients include proteins, oils, vitamins, minerals, and sugars, all of which are essential for human growth and development [20]. Insects are essentially animals, and their consumption is an act of ingesting food of animal origin, where the higher levels of nutrients are protein and fat. In comparison, edible insects have higher energy, protein, fat, polyunsaturated fatty acids, and cholesterol than animal flesh and a higher variety and content of trace elements than meat [4]. Some studies have shown that red meat consumption may increase the risk of stroke, diabetes, colon cancer, and lung cancer [21]. When compared with meat, insects seem to be more nutritious and healthier than food. Not only that, but insects are also still very diverse in terms of their nutritional content. Some statistics showed that insects are healthier than meat [22]. For this reason, insects are considered to be a meat substitute. For people who are over-nourished, eating insects may exacerbate the over-nutrition, but in cases of malnutrition, eating insects may be a good source of supplementary nutrients [22]. As an example, palm weevil larvae, which is one of the most famous edible insects in Asia and Africa. Studies have found that palm weevil (*Rhychophorus phoenicis*) larvae contain up to 66.3% total protein and 37.1% oil in dry weight. In addition, palm weevil larvae is a good source of potassium and phosphorus at 1025 and 658 mg/100 g, respectively [23]. The nutritional value of different varieties of palm weevil larvae can vary due to the different morphological types and growth conditions of palm weevil larvae [24]. However, there is no denying that palm weevil larvae are a high-quality source of protein, oil and trace elements. Some nutritionists have added fish oil and perilla seed to the diet of palm weevil larvae in order to improve the nutritional value of palm weevil larvae [25,26]. This resulted in elevated lipid and protein content, increased long-chain omega-3 polyunsaturated fatty acids, and increased essential amino acid and mineral content in palm weevil larvae. Undoubtedly, this has led to a substantial increase in the nutritional value of palm weevil larvae. According to recent reports, the palm weevil larvae have been used as a snack ingredient to enhance the protein and mineral content of snacks due to its high nutritional value [27]. In addition, the cookies with palm weevil larvae are more nutritious and at the same time have high sensory evaluation scores and acceptability [28]. The high nutritional properties of edible insects are gaining attention.

**Figure 1:**
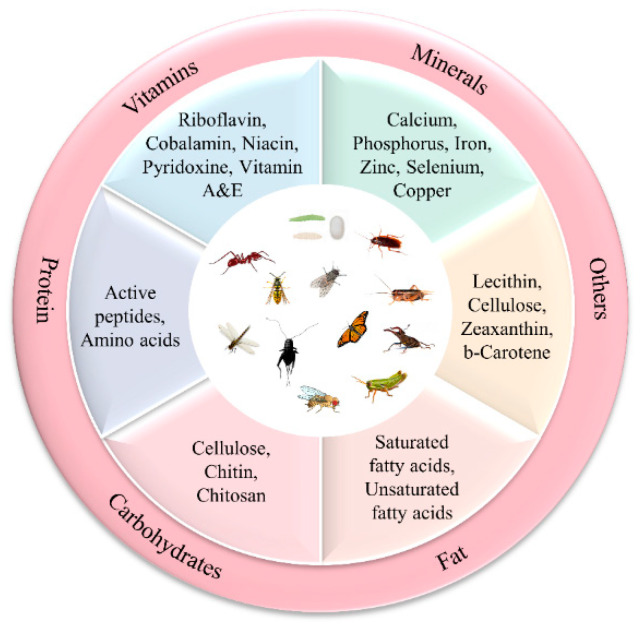
Nutrient composition of edible insects.

Recent studies on the nutritional composition of insects have shown that insects have attracted a great deal of attention from food scientists [29], nutritionists [4], and medical scientists [30,31] as a nutrient-rich food. Here, we summarized the nutritional value classification of edible insects. The nutritional composition of edible insects is depicted in Figure 1.

### 2.1. Proximate Composition of Matter of Selected Insects

**Figure 2:**
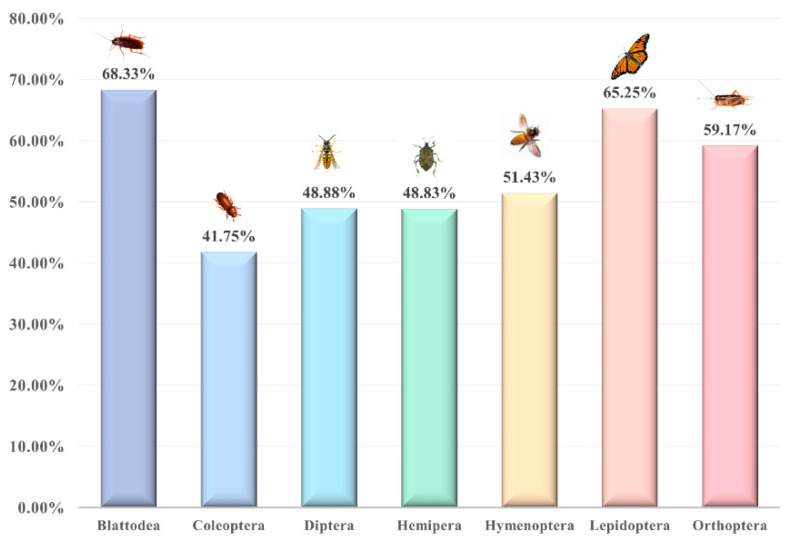
The protein content of edible insects of several common orders [10].

The approximate composition of the substances of 40 edible insects is given in Table 1. Different units are used for representation due to the different reference sources and the fact that unit conversions affect the completeness of the data. As can be seen from Table 1, the material composition of insects varies considerably between species. In dry matter, protein and fat are the more abundant substances. The protein content in the dry matter ranged from 6.25% to 80.26% and the fat content ranged from 2.2% to 43.0%. Moisture content is highest in the fresh weight of insects, while insects with less moisture content have a higher fat content [32]. It can also be seen from Table 1 that most insects contain less ash since they do not have the calcified skeleton that vertebrates have. The exception to this is *Musca autumnalis*, which has a high ash content of 63% by dry weight, compared to 1.2% in *Oxya chinensis* [33]. Figure 2 depicts the protein composition of different edible insect orders.

### 2.2. The Amino Acid Composition of Insect Proteins

Protein is an essential component of life and is in strong demand by humans. In the immune response, antibodies, which are essentially proteins, perform the immune function, and the majority of enzymes involved in biochemical reactions in animals are also proteins. Protein can even provide energy when the body needs it. The fact that insects are rich in protein has been widely reported [52,53]. The protein content of most insects ranges from about 35% to 60% dry weight or 10% to 25% fresh weight, which is already generally higher than that of cereals and legumes [54,55]. Species-wise, Orthoptera are generally higher in protein content, e.g., crickets, locusts, grasshoppers, etc. [56]. Therefore, insects are both feasible and necessary as a source of protein for humans [5]. Protein is made up of over 20 amino acids, but eight of them cannot be synthesized in the body and need to be taken in from outside to meet nutritional requirements. Interestingly, all of the essential amino acids can be found in insect proteins. The amino acid composition of 19 edible insects is listed in Table 2. It is clear that insect proteins contain not only a wide range of amino acids but also an abundance of essential amino acids.

### 2.3. Insect Fat

The fats in insects provide the body with a large amount of energy and essential fatty acids, and some specific fatty acids, such as linoleic acid and α-linolenic acid, play an important role in maintaining human health [59]. Therefore, insect fats are beneficial for human nutrition and health [60]. As the second most important nutrient in the body, insects can contain up to 43% of their dry weight in fat [43]. However, the fat content of insects varies greatly between species. For example, the average fat content of Coleoptera is 33.40%, while the average fat content of Orthoptera is only 13.41% [56]. In addition, studies have found that female insects are higher in fat, and that insect larvae and pupae have higher fat content in their periods [61]. The composition of fatty acids directly influences the nutritional quality of fats in food. In other words, the composition of saturated fat (SFA), monounsaturated fatty acids (MUFA), and polyunsaturated fatty acids (PUFA) in insect fat determines the nutritional quality of insect fat [62]. Unsaturated fatty acids are strongly associated with human health and disease, and the composition of fatty acids in the diet has been linked to diseases such as cancer, diabetes, and cardiovascular disease [63]. As can be seen from Table 3, most insect fats are rich in unsaturated fatty acids, which are very beneficial to humans [45]. It has been shown that insects contain a similar fatty acid composition to poultry and fish, but are higher in unsaturated fatty acids, especially polyunsaturated fatty acids [64,65]. However, there are exceptions. For example, in *Imbrasia ertli catepillars*, the saturated fatty acid arachidic is present up to 38% [55]. Nevertheless, the current research on insect-active substances is still on proteins, and more in-depth and extensive research is needed on insect fats [59].

### 2.4. Vitamins and Minerals

Vitamins and minerals are commonly found in insects, but vitamins are not usually synthesized in insects; they are mostly derived from insects that are enriched in their bodies through ingestion. Vitamins and minerals are essential in the metabolic processes of humans and animals, and their deficiency may have adverse health effects [67]. For example, growth retardation, anemia, inflammatory bowel disease, and other diseases associated with micronutrient deficiencies [68,69]. According to statistics, micronutrient deficiencies cause approximately one million premature deaths each year, demonstrating the need to improve food nutrition and that humans should not only pursue food production but also give due consideration to the nutrition of food [70]. Research has already shown that human micronutrient deficiencies can be addressed through the use of fisheries resources [71]. As insects contain a wide range of vitamins and minerals, most of the insects available are good sources of the vitamins and minerals that the human body needs [67]. Thus, we can consider dietary insects for the purpose of micronutrient supplementation.

So far, vitamin A, vitamin D_2_, vitamin D_3_, vitamin C, vitamin E, vitamin K thiamin, riboflavin, pantothenic acid, niacin, pyridoxine, folic acid, D-biotin, and vitamin B_12_ have been found in insects [49,56,61]. Of these, vitamin B_12_ is synthesized by bacteria and algae and accumulated in foods of animal origin. Therefore, the human body can obtain the required vitamin B_12_ through the intake of foods of animal origin [67,72]. Vitamin B_12_ has now been found in a variety of edible insects. A study investigated the levels of vitamin B_12_ in four insect species by immunoaffinity and ultra-high performance liquid chromatography, which is the first scientific report on the levels of vitamin B_12_ in edible insects [73]. The results showed that the vitamin B_12_ content of mealworm (*Tenebrio molitor* larvae), cricket (*Gryllus assimilis*), grasshopper (*Locusta migratoria*), and cockroach (*Shelfordella lateralis*) was 1.08 µg/100 g, 2.88 µg/100 g, 0.84 µg/100 g and 13.2 µg/100 g dry weight, respectively. These data suggest that vitamin B_12_ levels in insects do not appear to be high. However, some studies have shown relatively high levels of vitamin B_12_ in diving beetles and crickets, at approximately 89.5 and 65.8 µg/100 g dry weight, respectively [74]. House cricket is also considered a good source of B vitamins, such as thiamin, riboflavin, and folic acid, as they are found in abundance in house cricket [22,67]. Despite this, not all insects are rich in all types of vitamins and the vitamin content of insects is closely related to species, growing environment, food source, and developmental stage [75]. For example, Orthoptera and Coleoptera contain more folic acid than other insects [56]. In conclusion, edible insects may be rich in vitamins, but some species must be specifically selected to provide the required vitamins. It is also recommended to control the vitamin content of edible insects through feed. In Table 4, we list the vitamin composition and content of 12 insect species.

Insects are also good sources of minerals, especially iron and zinc, which may be of considerable nutritional importance [77]. Globally, micronutrient deficiencies continue to affect the health of 2 billion people, particularly iron, zinc and iodine deficiencies [78]. Micronutrient deficiencies are common in developing countries, especially among children and lactating women, with iron deficiency anemia, and iodine deficiency goiter being the main micronutrient deficiencies [79,80]. Table 5 summarises the mineral composition and content of 21 insects. Among the mineral species listed, it can be observed that the mineral composition and content of different insect species vary considerably, which may be related to the environment in which the insect is grown and the composition of its diet. However, Table 5 shows that minerals are present in insects, and in some insects, the levels of certain minerals are very high. Crickets (*onjiri mammon*) and termites (*oyala and agoro*), which are often used as food in Nigeria, are high in iron and zinc and relatively low in calcium, but insects still provide the calcium needed to meet the demand if they are used as the main food source [81]. A study assessed the mineral content of three edible cricket powders and found that 100 g of cricket powder could provide 14–22% of the recommended dietary nutrient supply of calcium. The cricket powder with the highest calcium content could contain 218 mg/100 g of calcium, which is comparable to the calcium content in foods such as tofu and salmon [15,82]. In addition, data from a study showed that grasshoppers, crickets, and mealworms contain much higher levels of chemically effective calcium, copper, magnesium, manganese, and zinc than beef brisket, with crickets, in particular, having a higher bioavailability of iron [83]. The above studies have shown that insects may be a good source of bioavailable iron. Nevertheless, insects are still rare as a source of micronutrients in the human diet. With more research on edible insects, they may become a major source of micronutrient intake for humans.

### 2.5. Other Components

**Figure 3:**
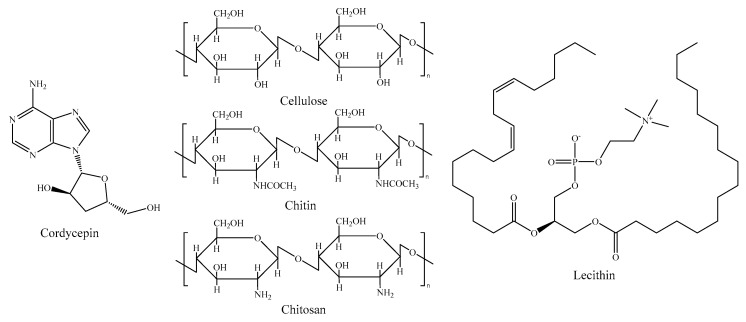
Structure of cordycepin, cellulose, chitin, chitosan, and lecithin [85,90].

In addition to the above, some insects contain carotenoids such as b-carotene, lutein, and zeaxanthin [50]. In *Acheta domestica*, the b-carotene content was up to 2.72 mg/kg. Carbohydrates in insects are mainly found in the exoskeleton and glycogen in the body [84]. The exoskeleton of insects consists mainly of a polymer of N-acetyl-D-glucosamine, the main source of cellulose, chitin, and chitosan [85]. *Tenebrio molitor* dry matter chitin content can reach 137.2 mg/kg [86]. And large amounts of chitin can be used to produce bioactive chitosan [87]. Consequently, insects are viable as a new source of chitin and chitosan [88]. Although the carbohydrate content of insects is low, polysaccharides from insects are also biologically active, e.g., silkworm pupae polysaccharides have immunomodulatory properties [52]. In some specific insects, there are also metabolites with strong biological activity, such as cordycepin in the insect fungus Chrysomelium [89]. Interferons, sex-attracting hormones, steroids, and lecithin have also been found in insects [85]. The nutrient content of insects varies according to species, stage of development, sex, environment, food composition, and processing methods [19,54]. By choosing the right insect species and controlling their growth environment and food composition, humans can raise insect products that meet human nutritional needs. Figure 3 illustrated the chemical structure formulae of several insect components.

## 3. Biological Functions of Insect Active Ingredients

**Figure 4:**
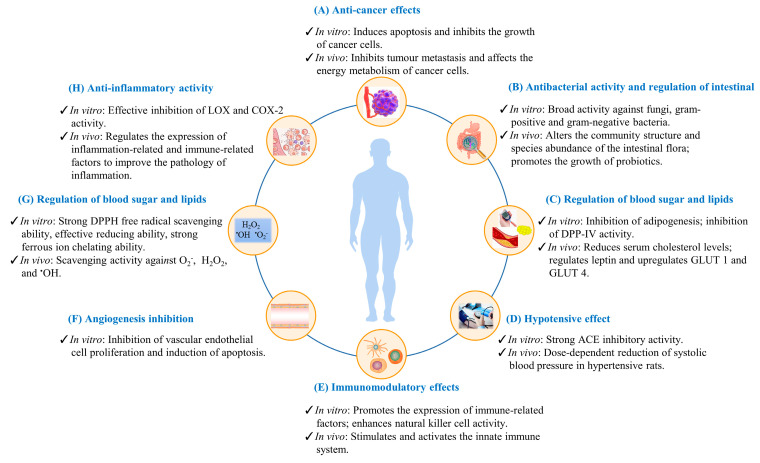
Biological functions of insect active ingredients and their potential mechanisms of action.

The active ingredients in edible insects have a variety of functional effects that are beneficial to human health. Broadly speaking, the effects of insects include tumor suppression, immunomodulation, antibacterial, antioxidant, anti-inflammatory, blood glucose and lipid regulation, blood pressure reduction, regulation of intestinal bacterial flora, and cardiovascular protection [15,91]. Table 6 summarized the functional effects of the insect active ingredients. The biological functions of insect active components and their probable mechanisms of activity are summarized in Figure 4. Here, we address several biological functions of insects in detail.

### 3.1. Anti-Cancer Effect

The anti-cancer effect is one of the most important effects of functional substances in insects and is therefore one of the most sought-after by researchers [85]. Many components of edible insects have anti-cancer activities. For example, active proteins, active peptides, trace elements, vitamins, chitosan, and other substances [13,161]. In vivo and in vitro studies have found that the active ingredients in the insects have inhibited cancers of the liver, stomach [96], colon [97], lung [93], breast [101], skin [161], and esophagus [162].

**Figure 5:**
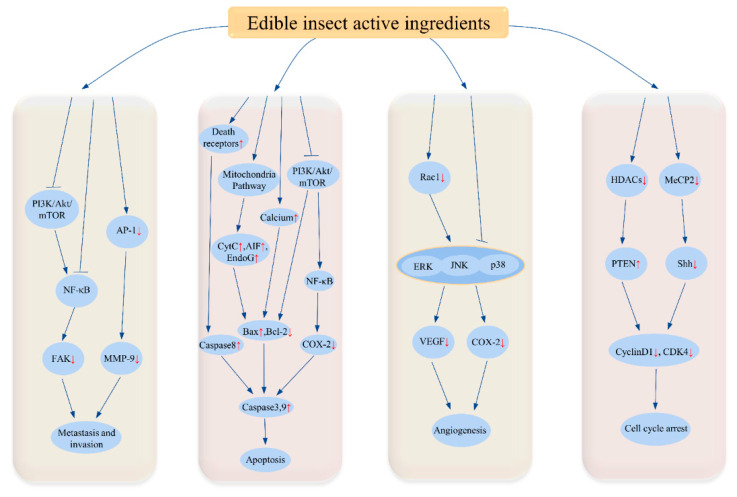
Potential anti-cancer mechanisms of action of insect active ingredients [171].

It is well known that the most characteristic feature of cancer is the abnormal proliferation and growth of cells in the body, and therefore the main function of most anti-cancer substances is to inhibit the abnormal proliferation and growth of cancer cells [163]. Some studies have shown that the active ingredients in insects have inhibitory effects on some cancer cell lines, such as human melanoma (SK-Mel-28) and primary pancreatic adenocarcinoma (Mia-PaCa-2) cells [92], NCI-H460 cells [93], B16F10 cells [94], human lung cancer A549 cell lines [95], human hepatoma cells [96], human colon cancer cells DLD-1 [97], human gastric cancer SGC-7901 cells [98], MGC-803 gastric cancer cells [99], U87-MG human glioblastoma cell lines [100], renal cell carcinoma cells [164], and breast cancer cells MCF-7 [101]. The anti-cancer functions of the insect active ingredients are summarized in Table 6, and their potential anti-cancer mechanisms of action are shown in Figure 5.

Among the many components with anticancer activity, protein-based substances are predominant. The anticancer activity of silkworm pupa proteins is outstanding. It was found that silkworm pupae protein hydrolysate could specifically inhibit the proliferation of human gastric cancer cells SGC-7901, induce apoptosis, and block the cell cycle in the S phase [98]. A selenium-rich amino acid from silkworm pupae significantly inhibited the viability of human hepatoma SMMC-7721 cells, induced changes in cell morphology and cycle, and caused apoptosis [96]. Recent studies have also shown that silkworm pupae proteins and their hydrolysates can inhibit the growth and reproduction of cancer cells and promote apoptosis by affecting the mitochondrial function of cancer cells and thus their energy metabolism [97,99]. The silkworm pupae protein also exerts anti-cancer effects by down-regulating the expression of IL-6, IL-1β, and TNF-α in human breast cancer cells, as well as causing biochemical changes in lipids, proteins, and nucleic acids [101]. Some active peptides from insects also show cytotoxicity against cancer cells, such as D-9-mer peptides from beetles [165], CopA3 peptides from *Copris tripartitus* [166], antimicrobial peptides based on insect defensin [167], and the antimicrobial peptide harmoniasin of ladybird beetles [168]. These anti-cancer peptides are highly efficacious, easily modified and synthesized, less resistant to target cells, and largely non-toxic to mammalian erythrocytes and macrophages [167,169,170]. In addition, a recombinant protein from *amblyomma cajennense*, amblyomin-X, was found to induce tumor cell death by regulating cell cycle-related genes and targeting the ubiquitin-proteasome system [92].

Bee venom has traditionally been used to treat rheumatism and skin diseases. However, numerous studies have found that bee venom can inhibit cancer cell growth by promoting apoptosis in prostate and ovarian cancer cells [172,173] and can also induce caspase-dependent and caspase-independent apoptotic cell death by activating the Ca^2+^-regulated intrinsic death pathway in human bladder cancer cells [174]. In addition, bee venom also inhibited lung cancer cell lines A549 and NCI-H460, and bee venom-induced apoptotic death in lung cancer cells by enhancing death receptor 3 expression and inhibiting the NF-κB pathway [93]. The main component of bee venom is melittin, which has a variety of biological effects [175], and a great deal of research has focused on the anti-tumor effects of melittin and its role in cancer treatment [176]. A review has discussed the use of bee toxins in cancer therapy. The article summarized the possible mechanisms of the anticancer effects of bee toxins, mainly through the induction of apoptosis, inhibition of tumor metastasis and invasion, arrest of the cell cycle, and blocking of angiogenesis [171].

Insect fats and sugars also have anticancer activity. It was found that mealworm larvae oil inhibited human hepatocellular carcinoma (HepG2) and colorectal adenocarcinoma (Caco-2) cells, with the potential anti-cancer mechanism being apoptosis via activation of the death receptor pathway for Caspase-8, -9, and -3 [177]. A polysaccharide-protein complex from *Scolopendra subspinipes mutilans L. Koch* was found to have anti-tumor activity in tumor-bearing mice. This polysaccharide-protein complex inhibits tumor growth in mice by downregulating the AA-metabolic pathway in TAMs [178]. It was also found that glycosaminoglycans from dung beetles could enhance the extracellular matrix by increasing TIMP-2 activity and adhesion activity to collagen, thereby inhibiting extracellular matrix changes and thus tumor cell invasion and progression [94].

Some secondary metabolites in insects also exhibit anticancer effects. *Cordyceps cicadae* are widely used in China for medicinal purposes. The compounds cordycepin, ergosterol peroxide, cordycecin, and beauvericins in *crdyceps cicadae* have been reported to have anticancer effects on a variety of cancer cell lines, and the ethanolic and aqueous extracts also have anticancer properties [179]. It has been shown that ergosterol peroxide in cordyceps cicadae inhibits renal cell carcinoma cells in vitro through a variety of mechanisms, including inhibition of cancer cell growth, migration, and invasion; inhibition of the cell cycle; weakening of β-catenin pathways, and triggering apoptosis [164].

The above studies show that insects are a viable source of anticancer active ingredients, whereby highly active, less toxic, and effective anti-tumor drugs can be developed, thereby promoting human health.

### 3.2. Antioxidant Activity

Oxygen is very important for animals and is essential for human cellular metabolic processes. However, reactive oxygen species (ROS), such as superoxide anions (O_2_^−^), hydrogen peroxide (H_2_O_2_), and hydroxyl radicals (^•^OH), produced during cellular metabolism can cause serious damage to cells if they accumulate in excess and reach high concentrations [180]. Oxidative stress is associated with many diseases such as cancer, heart disease, arthritis, and aging [181]. A variety of active ingredients in edible insects have been reported to possess antioxidant activity. It was found that extracts obtained from insects by different methods, hydrolysis products obtained using various enzymatic treatments, various peptides isolated and purified, and substances such as chitosan all had antioxidant activities of different intensities (Table 6).

A study examined the in vitro antioxidant activity of water- and fat-soluble extracts of 12 commercially available insects. The water-soluble extracts from grasshoppers, silkworms, and crickets were found to have the highest antioxidant activity, five times that of fresh orange juice, while the fat-soluble extracts from the silkworm, evening cicada, and African caterpillars had the highest antioxidant activity, up to twice that of olive oil [182]. Orange juice and olive oil are known to be functional foods that regulate antioxidant activity in the body due to their high antioxidant activity [183,184]. So, it is theoretically feasible to use insects to develop antioxidant-functional foods. The aqueous extract of housefly larvae prepared using the decoction method was reported to have significant antioxidant activity, with a DPPH radical scavenging activity of 75.4% at a level of 5 mg/mL [185]. In addition, the extracts obtained from *Acheta domesticus* and *Tenebrio molitor* using ultrasound-assisted extraction and pressurized liquid extraction also exhibited antioxidant activity. Using GC-MS characterization, it was found that the substances exhibiting antioxidant activity were mainly the total phenolic compounds in the extracts [186].

Currently, many studies have focused on the antioxidant activity of insect proteins or peptides. In vitro, antioxidant activity experiments were carried out using housefly larvae protein hydrolysis products obtained by albumin hydrolase and neutral protease enzymatic digestion. The results showed that both protein hydrolysates exhibited superoxide and hydroxyl radical scavenging activity and that their antioxidant activity was proportional to the concentration of the hydrolysate [187]. Another study prepared silkworm larvae protein hydrolysate using gastrointestinal enzymes, which also exhibited relatively high DPPH radical scavenging activity (IC50 = 57.91 µg/mL) and ferrous ion chelating ability (IC50 = 2.03 mg/mL) [188]. DPPH radical scavenging experiments on male silkworm hydrolysate peptides revealed that peptides with molecular weights greater than 30 kDa were the main components with antioxidant functions and that this fraction of insect peptides had good stability, with antioxidant activity remaining at 80% after simulated gastrointestinal digestion [189]. Similarly, the hydrolysis of whole crickets using calcineurin not only reduces the allergenicity of cricket proteins, but the resulting bioactive peptides also have antioxidant activity. The active peptide was found to have maximum bioactivity potential at 60–85% hydrolysis [190]. A peptide fraction from a cricket (*Gryllodes sigillatus*) hydrolysis product showed outstanding antioxidant potential with a semi-inhibitory concentration of 10.9 µg/mL for DPPH radical scavenging activity [191]. In addition, a potent antioxidant peptide purified from a weaver ant (*Oecophylla smaragdina*) with a CTKKHKPNC sequence had a semi-inhibitory concentration of 48.2 µmol/L for DPPH and 38.4 µmol/L for ABTS [192].

In addition to the above components, insect chitosan is also a good source of antioxidant active substances. Chitosan isolated from housefly larvae (*Musca domestica*) has a strong scavenging capacity for DPPH free radicals, as well as an effective reducing capacity and a strong chelating capacity for ferrous ions, and its DPPH free radical scavenging capacity is stronger than that of ascorbic acid, with a semi-inhibitory concentration of approximately 0.373 mg/mL [193]. Another study showed that chitosan from blowfly larvae (*Chrysomya megacephala*) exhibited good antioxidant activity with a semi-inhibitory concentration of about 1.2 mg/mL of DPPH radical scavenging activity, and the molecular weight of this chitosan (501 kDa) was significantly lower than that of commercial chitosan (989 kDa) [194].

A recently published article reviews the main findings on the antioxidant properties of edible insects. This article summarized the relationship of edible insects to oxidative stress in various in vitro and cellular models as well as in animal studies, and illustrated the feasibility of developing antioxidant-enabled products from edible insects [195]. However, much of this work has been carried out in vitro and in animal models, and clinical trials are needed to support the antioxidant properties of insect products for human health.

### 3.3. Antibacterial Activity and Effect on Intestinal Microorganisms

**Figure 6:**
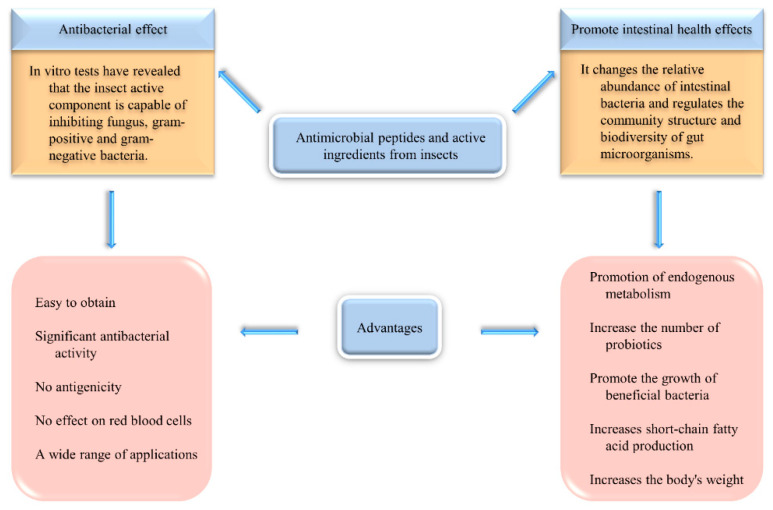
The antimicrobial activity of insect active substances and their effects on intestinal bacteria, as well as the advantages of action effect.

Insects have a variety of substances with antibacterial activity, including insect proteins and peptides, insect oils, chitosan, and chitin, among which the most representative are antibacterial peptides. Since the first discovery of insect antimicrobial peptides in *Hyalophora cecropia* in 1980, more than 150 species have been identified [196]. Antimicrobial peptides are generally a class of peptides produced by a variety of species, such as insects, other animals, microorganisms, etc. They have short peptide chains, are heat stable, and have no drug sensitivity or effect on eukaryotic cells [197]. It has been shown that antimicrobial peptides can exert a range of antimicrobial activities by isolating key growth nutrients, permeabilizing bacterial membranes, and other related mechanisms [198]. Antimicrobial peptides are diverse, can be found in all organisms, and interact with intestinal microorganisms [199]. Figure 6 depicted the antimicrobial activity of insect active compounds, as well as their influence on intestinal bacteria and the advantages of the action effect.

A wide variety of antimicrobial peptides have been obtained from insects, and many studies have purified and identified antimicrobial peptides from insects and verified their antimicrobial activity. For example, papain peptides isolated from swallowtail butterflies (*Papilio xuthus*) have broad activity against fungal, gram-positive, and gram-negative bacteria and no hemolytic activity against human red blood cells [200]. Antimicrobial peptides purified from the plasma of silkworm (*Bombyx mori*) larvae were found to have a significant growth inhibitory effect on different Gram-positive and Gram-negative strains of bacteria, which led to the use of antimicrobial peptides from silkworm larvae as an alternative to antibiotic treatment in this study [201]. Eight peptides purified from the lymphatic blood of the greater wax moth (*Galleria mellonella*) were also examined for their antibacterial activity. These peptides were found to have antibacterial activity against gram-negative and gram-positive bacteria, yeasts, and filamentous fungi, with the defensin-like peptide being the most effective, inhibiting the growth of sensitive bacteria at a concentration of 1.9 µM [202]. In another study, royalisin (a 5.5-kDa antimicrobial peptide) isolated from royal jelly showed antimicrobial activity against fungi, gram-negative and gram-positive bacteria, and its antimicrobial activity was found to be related to the disulfide bond in the peptide [107]. In particular, a novel antimicrobial peptide (amino acid composition Gly-Gly-Gly-Gly-Gly-His-Leu-Val-Ala) from tasar silkworm (*Antheraea mylitta*) was effective in killing urinary tract-associated MDR E. coli. The antimicrobial peptide interacts with the lipid fatty chain of the 1-palmitoyl-2-oleoyl-phosphoethanolamine bilayer for bacterial inhibition and is non-antigenic and has no effect on erythrocyte membranes [110]. Interestingly, the simultaneous action of different antimicrobial peptides from insects can enhance the antimicrobial effect of the antimicrobial peptides, thus allowing a better antimicrobial effect to be achieved with a smaller amount of antimicrobial peptide use. Further studies have shown that this interaction through the combination of antimicrobial peptides can be used to treat gram-negative bacterial pathogens that have become resistant to common antibiotics [203]. In addition, chitin and chitosan from silkworm pupae [108], chitin from cockroaches [102], secretions from forest caterpillars (*Calosoma sycophanta*) [106], defensins from insects [204], and silkworm pupa oil and silk [105,205] also have antimicrobial activity.

Previously, there was no evidence directly demonstrating the relationship between insect antimicrobial peptides and gut microbes, but the community structure of gut bacteria is closely related to the structure of the diet. Several studies in recent years have gradually identified the effects of insect consumption on the gut flora. Studies in animals have found that insect consumption can influence the community structure and biodiversity of gut microbes in some animals. An experiment using rats has shown that feeding insect protein to rats instead of meat can affect endogenous metabolism by altering the diversity of the gut microbiota [206]. Similarly, the addition of silkworm pupa oil to sheep feed can alter the relative abundance of sheep intestinal bacteria, thereby reducing sheep intestinal methane emissions and increasing sheep body weight [205]. In addition, feeding black soldier flies (*Hermetia illucens*) to rainbow trout and laying hens also altered the structure of the intestinal flora and species abundance [151,152]. In addition, experiments on humans have been reported. One study used a double-blind, randomized crossover trial to investigate the effects of edible cricket intake on the gut microbiota of healthy adults. The results found a 5.7-fold increase in intestinal probiotics (bifidobacteria) in participants who consumed cricket powder, and it was also hypothesized that cricket powder could improve gut health and reduce inflammation in the body [153]. In another study, the effect of *Tenebrio molitor* flour on human intestinal flora was investigated in an in vitro digestive simulation model [207]. *Tenebrio molitor* flour was found to promote the growth of *Bacteroidaceae* and *Prevotellaceae* and the production of short-chain fatty acids in the intestine, which are closely related to the metabolism of substances in the human body.

### 3.4. Anti-Inflammatory Activity

The inflammatory response is the body’s protective behavior in response to abnormalities in the body caused by external or internal adverse factors, and it has been revealed that the inflammatory response is involved in a wide range of biological processes in the organism [208]. The presence of components with anti-inflammatory activity in some edible insects was found, and the anti-inflammatory function of edible insects is closely related to other active functions [209].

Firstly, most of the substances with anti-inflammatory activity in insects are protein-based. For example, hydrolyzed peptides obtained by heat treatment from three edible insects (*Gryllodes sigillatus, Tenebrio molitor, Schistocerca gragaria*) all exhibited anti-inflammatory properties, with the peptides from *Gryllodes sigillatus* showing strong lipoxygenase (LOX) and cyclooxygenase-2 (COX-2) inhibitory activities (semi-inhibitory concentration values of 0.13 and 0.26 µg/mL, respectively) [125]. In another study, the anti-inflammatory activity of peptide fractions from edible insects was also characterized by their inhibitory activity against LOX and COX-2, and peptide fractions from *Tenebrio molitor*, *Schistocerca gregaria*, and *Gryllodes sigillatus* were found to be effective in inhibiting the activity of LOX and COX-2. The peptides from *Schistocerca gregaria* showed the most significant inhibitory effect (semi-inhibitory concentrations of 3.13 µg/mL and 5.05 µg/mL respectively) [191]. Both studies found that heat treatment enhanced the activity of these insect antioxidant peptides. In addition, insect peptides obtained using enzymatic digestion also exhibited anti-inflammatory activity. The anti-inflammatory activity of three edible insects (*Tenebrio molitor, Gryllus bimaculatus, Bombyx mori*) hydrolysates assessed by macrophage production of nitric oxide found that *Bombyx mori* hydrolysates exhibited significant anti-inflammatory activity regardless of the hydrolysis method used [210]. In cellular assays, some insect active ingredients have also shown anti-inflammatory activity. For example, it was found that the venom of the ectoparasitoid wasp (*Nasonia vitripennis*) has anti-inflammatory effects on mammalian cell lines and that its mechanism of action is through the inhibition of NF-κB signaling in mammalian cells, which in turn affects the expression of inflammation-associated and immune-associated factors [124]. Another study also evaluated the anti-inflammatory activity of hemolymph from *Lycorma delicatula* in lipopolysaccharide-induced RAW264.7 cells and indicated its feasibility as a preventive measure against inflammatory damage in skin tissues [211].

In addition to this, in vivo studies have also shown that peptides, sugars, and other substances from insects have anti-inflammatory activity. A study has investigated the anti-inflammatory effects of glycosaminoglycans from crickets (*Gryllus bimaculatus*) using a rat model of chronic arthritis. It was found that this glycosaminoglycan exerted anti-inflammatory effects through modulation of pro-inflammatory cytokines and improvement of histological pathology, with anti-inflammatory effects comparable to those of anti-inflammatory drugs [120]. In addition, the protein extract of housefly maggots (*Musca domestica*) was found to be effective in inhibiting various pro-inflammatory responses in experimental atherosclerotic lesions in vivo, and also inhibited lipopolysaccharide-induced expression of TNF-α, IL-1α, and MCP-1 in macrophages in vitro [121]. In China, an edible black ant (*Polyrhachis dives*) is known for its kidney-protective and anti-inflammatory properties. Thirteen non-peptide nitrogenous compounds were isolated from the black ant using ethanol extraction and chromatographic separation, and biological studies have shown that some of these compounds have good anti-inflammatory and immunosuppressive activity [123].

A recently published review discusses the effects of bioactive components from different edible insects on inflammation and its associated complications (colitis and arthritis) and cancer [209]. It was concluded that the use of insect-active ingredients as drug molecules for the treatment of various inflammation-related diseases is promising. In the meantime, however, the toxicological characteristics and targets of these active substances need to be further elucidated to develop functional foods or pharmaceuticals with beneficial effects on human health in the near future.

### 3.5. Regulation of Blood Lipids and Blood Glucose as Well as Anti-Obesity and Anti-Diabetic Activity

Blood glucose and blood lipids are two important health-related indicators of blood, and high or low blood glucose and lipids can directly affect the normal metabolism of the body and the development of various diseases, such as obesity and diabetes [212]. According to statistics, various health problems caused by obesity and diabetes have become major contributors to human mortality worldwide (WHO, 2000). The prevalence of type 2 diabetes is increasing every year, while obesity has also been found to contribute to the development of diabetes [213]. Excitingly, peptides have been discovered and used as potential therapeutic agents for diabetes and obesity [214]. As a consequence, researchers are actively investigating the active substances in edible insects that regulate blood lipids and blood glucose, and in this way, they are exploring and developing functional substances with anti-obesity and anti-diabetic activity.

Some substances from insects have been found to have a regulatory effect on lipid metabolism and blood lipid levels in the body, which in turn has an anti-obesity effect. These include *Tenebrio molitor* larvae [215], *Allomyrina dichotoma* larvae [216,217], chitooligosaccharides from *Clanis bilineata* [218], glycosaminoglycans from crickets (*Gryllus bimaculatus*) [130], and silkworm pupae powder and pupae polypeptides [132,134]. In vivo studies of ethanol extracts from *Tenebrio molitor* larvae found that they reduced lipid accumulation and triglyceride levels in mature adipocytes and induced phosphorylation of adenosine monophosphate (AMP)-activated protein kinase and mitogen-activated protein kinase, leading to weight loss in obese mice [215]. The anti-obesity effects of *Allomyrina dichotoma* larvae extracts have been studied relatively extensively. In vitro studies using 3T3-L1 cells revealed that *Allomyrina dichotoma* larval extracts could inhibit adipogenesis in 3T3-L1 cells through a potential mechanism of action by downregulating the expression levels of mRNA and related proteins [217]. In vivo studies found that *Allomyrina dichotoma* larvae extract reduced serum levels of oil triglycerides and leptin in obese mice and reduced weight gain, organ weight, and adipose tissue volume in a dose-dependent manner [216]. Glycoconjugates from edible insects exhibit similar hypolipidemic effects. The chitooligosaccharides from *Clanis bilineata* were reported to have significant hypolipidemic effects in rats, as evidenced by the reduction in plasma triacylglycerol (TG), total cholesterol (TC), and plasma low-density lipoprotein cholesterol (LDL-C) levels [218]. Similarly, glycosaminoglycans from *Gryllus bimaculatus* can affect serum levels of phospholipids, cholesterol, and glucose in rats by regulating genes related to lipid metabolism [130]. A recently published review has illustrated that the active substances in silkworm pupae have a variety of biological functions, which highlights the hypolipidemic effects of the active components of silkworm pupae [219]. Silkworm pupa powder and silkworm chrysalis peptides can exert hypolipidemic effects by regulating the body’s lipid metabolic processes and by inhibiting fat formation in preadipocytes, and by reducing lipid accumulation and adipocyte size to improve obesity [132,134].

The regulation of blood glucose is usually related to insulin levels, dipeptidyl peptidase 4 (DPP-IV) activity, and glucose transporter (GLUT) levels in the body [220]. Several studies have found that whole crickets (*Gryllodes sigillatus*) [190], lesser mealworm (*A. diaperinus*) protein [221], and water extract of housefly larvae (*Musca domestica*) [185] have inhibitory activity against DPP-IV. In particular, water extract of housefly larvae showed semi-inhibitory concentrations of up to 3.52 mg/mL against DPP-IV. In addition, studies using a mouse 3T3-L1 adipocyte cell line found that fibrin hydrolysate from silkworms accelerated glucose metabolism and glycogen conversion in cells and increased GLUT 1 on the cell surface while enhancing translocation of GLUT4 [133]. Another study showed that silk protein hydrolysate increased glucose uptake through the upregulation of GLUT 4 and reduced fat accumulation through the upregulation of leptin [131]. The ethanolic extract from *Oxya Chinensis Sinuosa* also slows carbohydrate digestion and glucose absorption by inhibiting the activity of carbohydrate digestive enzymes, thereby reducing postprandial hyperglycemia caused by dietary carbohydrates [222].

The above research gives us a strong reminder of the promise of developing drugs with lipid and blood glucose modulating properties through the use of edible insects, which could be a boon to diabetics and obese people.

### 3.6. Hypotensive Effect

Hypertension increases the risk of cardiovascular disease and is more common in the elderly and obese people. In the body, the angiotensin-converting enzyme (ACE) is a key enzyme in the regulation of blood pressure. It catalyzes the hydrolysis of angiotensin I to the vasoconstrictor angiotensin II, which leads to vasoconstriction and consequently increases blood pressure [223]. And studies have found that the various synthetic ACE inhibitors used in pharmaceuticals can cause serious side effects [224]. The search for natural ACE inhibitors is therefore essential. Studies on the hypotensive function of edible insects and their components have also focused on ACE inhibition.

The presence of hydrolysis products and peptides with hypotensive activity in the larvae or pupae of silkworms has been reported in the literature [136,225]. For instance, hydrolysates of silkworm larvae prepared by gastrointestinal enzymes showed strong ACE inhibition in vitro with an IC50 of 8.3 µg/mL [188]. The fractions in the hydrolysate of silkworm pupae protein produced using acid protease also had ACE inhibitory effects, with albumin being the most potent inhibitor [226]. A peptide hydrolysate of silkworm pupae with a molecular weight of less than 5000 Da prepared by ultrafiltration not only exhibited ACE inhibitory activity in vitro, but also reduced systolic blood pressure in spontaneously hypertensive rats in a dose-dependent manner, and did not affect blood pressure in normal rats [135]. In addition, the enzymatic products obtained from the hydrolysis of cotton leafworm (*Spodoptera littoralis*) by pepsin, trypsin, and a-chymotrypsin all showed strong ACE inhibitory activity [227,228]. In addition, the enzymatic peptide from *Gryllodes sigillatus*, *Oecophylla smaragdina*, also has the function of inhibiting ACE activity in vitro [190,192]. These edible insect active components’ ACE inhibitory effect suggests that edible insects are a potential source of active ingredients for blood pressure-lowering medications. Moreover, given that they are natural, these compounds may be safer for people.

### 3.7. Immunomodulatory Effects

Because of the immunological complexes in their bodies, insects have robust immune systems [229]. In addition, the majority of insects are protein-rich, and certain active peptides produced during protein hydrolysis have immunomodulatory properties [230]. Insect active ingredients have been demonstrated to have immunomodulatory effects [122]. For instance, a novel active peptide with the amino acid sequence Asp-His-Ala-Val was extracted from silkworm larvae and was found to have immunomodulatory effects by inducing the production of immune-related proteins. Its molecular weight is 441.06 Da. The relevant immune-related factors include IL-6, IL-12, NF-κB, cyclin D1, and cell cycle protein-dependent kinase 4 [128]. Additionally, a peptide from the blow fly Calliphora vicina known as alloferon was discovered to stimulate natural killer lymphocytes in vitro experiments. Alloferon was then discovered to have antiviral and anti-tumor properties in vivo experiments in mice, and these effects are probably related to the immune system in mice [119]. In addition to this, bee venom phospholipase A2 can induce T-helper type 2 cell type responses and group 2 innate lymphoid cell activation via enzymatic cleavage of membrane phospholipids and IL-33, suggesting that bee venom can stimulate and activate the innate immune system in mice [126]. Similarly, a polysaccharide from the silkworm was shown to activate the innate immunity of RAW264 cells and penaeid prawns, and in vivo experiments demonstrated that activation of innate immunity was effective in preventing vibriosis in prawns [127]. The immune system is implicated in many human diseases and the immunomodulatory effects of edible insects may be a route of action for other active functions [230].

### 3.8. Angiogenesis Inhibition

Angiogenesis is a complex process involving multiple cells and multiple molecules and is relevant to the development and treatment of many diseases in the human body, for example, tumor therapy [231], renal disease [232], cardiovascular disease [233], etc. A study used vascular endothelial growth factor (VEGF) to induce angiogenesis in human umbilical vein endothelial cells and four bee products to study their inhibitory effect on angiogenesis. The outcomes demonstrated a potent inhibitory impact of Chinese red propolis and caffeic acid phenethyl ester on VEGF-induced angiogenesis [139]. A troponin I-like molecule from *Haemaphysalis longicornis*, is considered to be a potent inhibitor of angiogenesis. This is due to its ability to significantly inhibit capillary formation in human vascular endothelial cells (HUVEC) in vitro at a semi-inhibitory concentration of 18.95 nM [137]. Similarly, haemangin from *emaphysalis longicornis* can disrupt angiogenesis and wound healing by inhibiting vascular endothelial cell proliferation and inducing apoptosis [141]. The information above may suggest that the consumption of insects may have the potential for the prevention and treatment of angiogenesis-related human diseases. However, substances from insects with angiogenesis-inhibiting effects appear to be mostly components of insect venom, and therefore the safety of these active substances is something that should be considered first in the future if applied to the treatment of human diseases.

### 3.9. The Therapeutic Effects of Several Common Diseases

**Figure 7:**
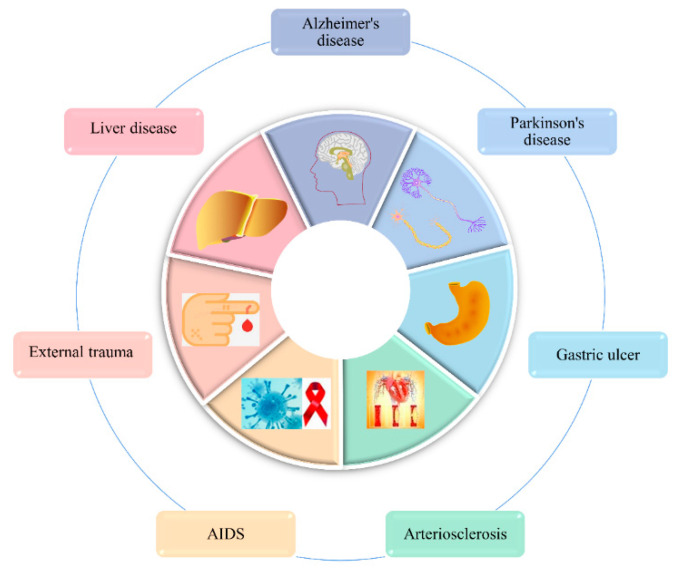
Therapeutic effects of edible insects on several common diseases.

The consumption of insects has been found to have a therapeutic or ameliorative effect on several common diseases. Figure 7 shows the therapeutic effects of edible insects on several ailments.

#### 3.9.1. Alzheimer’s Disease (AD) Therapy Effects

It was shown that giving silkworm pupa powder to adult male AD rat models dramatically lessened memory loss and decreased hippocampus neuronal density. By improving cholinergic function and exerting neuroprotective benefits by decreasing oxidative stress in rats, the drug’s possible mechanism of action is to improve cognitive performance [156]. Additionally, a study that used silkworm pupae to create an AD vaccine was discovered to enhance memory and cognitive function in AD mice in an in vivo investigation [157]. This implies that silkworm pupae may one day be used in medicine as an ingestible insect for treating AD.

#### 3.9.2. Therapeutic Effects of Parkinson’s Disease (PD)

A study evaluated the anti-inflammatory effects of bee venom in a 1-methyl-4phenyl-1,2,3,6-tetrahydropyridine (MPTP)-induced PD mouse model. Bee venom was found to be neuroprotective, attenuating the activation of microglial cell responses and reducing neuroinflammation in a model of MPTP-induced Parkinson’s disease [234]. In addition, bee venom was found to inhibit Jun activation, thus effectively protecting dopaminergic neurons from the toxicity of MPTP [235]. Further research has revealed that apamin, a component of bee venom, can mimic the protective effect of bee venom treatment on dopaminergic neurons, and in vivo experiments have shown that apamin, together with other components, can enhance this protective effect and work together to improve PD [155]. The findings of these studies have important implications for the treatment of PD and can also suggest that insect components have a functional role in the treatment of PD.

#### 3.9.3. The Therapeutic Effect of Gastric Ulcers

Protective effect of silkworm pupa oil against hydrochloric acid/ethanol-induced gastric ulcers. Firstly, silkworm pupa oil reduced gastric ulcer area and gastric secretion and increased gastric pH. Secondly, it increased serum SOD, CAT, GSH-Px, SST, and VIP levels and reduced IL-6, IL-12, TNF-α, IFN-γ, MTL, and GT levels. In conclusion, the treatment of silkworm pupae oil can protect against gastric ulcers by reducing oxidative damage and inflammation in mice [159] α.

#### 3.9.4. Therapeutic Effects of Atherosclerosis

The active ingredients of silkworm larvae and pupae can be used to treat many cardiac and neurological disorders. Results of studies using silkworm pupae extracts on their effects on hyperlipidemia and atherosclerosis found a significant reduction in hyperlipidemia in the treatment group and a reduction in the size of atherosclerotic plaques on histopathology. The study concluded that the therapeutic effect of silkworm pupae extract on atherosclerosis may be due to its antioxidant and hypolipidemic functional effects [117].

#### 3.9.5. Anti-HIV

AIDS, caused by the Human Immunodeficiency Virus (HIV), has always been a serious health risk. However, there is still no vaccine available to control and prevent HIV. Nevertheless, some studies have shown that active ingredients from honeybees and scorpions may have inhibitory effects on HIV. For example, the uptake of bee venom peptides by HIV-infected cells resulted in a reduction in HIV gene expression and replication. Limiting HIV replication has a positive effect in the fight against AIDS and this may help in the treatment of AIDS [154].

#### 3.9.6. Therapeutic Effects of External Trauma

Honey has long been used as a healing remedy for wounds, burns, and ulcers [145]. The results of randomized clinical trials have shown that honey can be used as a wound healing agent because of its antibacterial activity to protect wounds from infection, its anti-inflammatory activity to reduce wound edema, and its ability to stimulate the growth of granulation and epithelial tissue, thereby accelerating wound healing [236]. Another study also found that royal jelly protein 1 in honey promoted an increase in cytokines (TNF-α, IL-1β, and TGF-β) and metalloproteinase-9 (MMP-9) in human keratin-forming cells. This suggests that honey activates keratinocytes, demonstrating its ability to accelerate wound healing [146].

### 3.10. Other Functions

In addition to the functional effects described above, some other functional activities have been reported for insects and their active ingredients. For example, some insects with high protein content, including silkworms, can be used as anti-fatigue agents to improve muscle strength in humans or animals after exercise [148,149]. Exercise supplements can be developed accordingly to improve fatigue from exercise [15]. Fermented cricket powder has been found to promote hair growth. As a hair growth promoter, fermented cricket powder may be a promising remedy for hair loss [160]. Other functions of insect consumption include improvement of facial skin wrinkles [150], reduction in liver damage [116], alcohol detoxification [158], anti-apoptotic effects [142], and anti-genotoxic effects [144]. In addition to this, some of the functional substances in edible insects are also known to be anti-radiation, improve osteoporosis, improve anemia and improve memory [237]. The functions of the insects and their active ingredients are summarised in detail in Table 6.

## 4. Factors to Consider in the Consumption of Edible Insects

Human use of insects as food has become commonplace, with nearly two billion people worldwide using insects as food [238]. The use of insects to develop drugs for human diseases is still challenging, but solutions are gradually being sought [13]. Many studies have now shown that insects are feasible for use as medicines in the treatment of diseases [12,239,240]. Although edible insects serve many useful purposes, their potential safety and other potential negative effects must be carefully evaluated whether utilized as food or medication.

### 4.1. Acceptance of Insects

**Figure 8:**
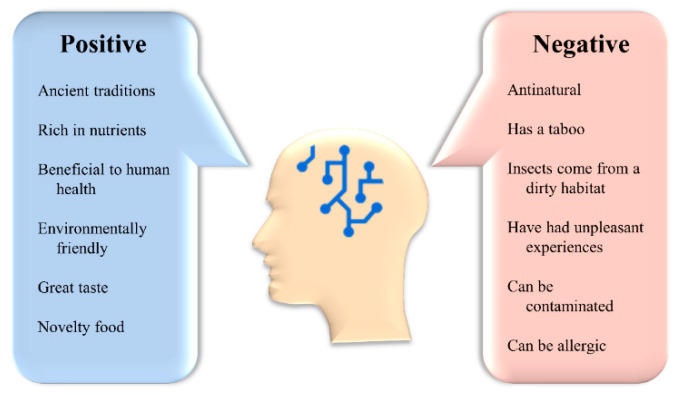
Different attitudes towards edible insects as food.

The first is consumer acceptance of insect food, which is the first thing that determines whether consumers accept insect food or not [241]. However, for Westerners, there seems to be a reluctance to try eating insects. Despite the availability of insect delicacies in some restaurants, most consumers are still reluctant to eat insects [242]. The study found that the two main barriers to insect consumption for European consumers are food neophobia and disgust [242,243]. People with food neophobia may accept the consumption of insect food in the future, but attitudes towards insect food among people with an aversion to it will be difficult to change. Men, according to studies, are more responsive to insect goods than women, and young individuals are less adverse to consuming insects than older ones [244]. In response to the foregoing, it was proposed that to increase the acceptability of insect food, the flavor, texture, and appearance of edible insects may be altered to fit the demands of different groups of people, hence lowering aversion to edible insects in certain groups [243]. Figure 8 shows the various opinions about edible insects as food.

### 4.2. The Difference between Wild and Farmed Insects

Edible insects were mostly wild in earlier times, and ancient humans were very limited in raising and eating them. Only a few insect species were raised by humans for long periods of time and could be used for food, such as *Apis mellifera* and *Bombyx mori* [245]. Due to the curiosity about wild foods, many people believe that wild insects are more nutritious and have better health benefits. But this is not necessarily the case. Termites are an insect used as food in Africa and North Gondwana, Asia, and some studies have found that the difference in minerals between wild harvested termites and commercially available termites is significant [246]. The data showed that the amount of manganese in wild termites was 50–100 times higher than the concentration detected in other insects. From a safety point of view, wild insects may have excessive levels of heavy metals due to the complex and harsh growing environment [247]. This is not good for insects as a food source. In addition to this, wild harvested edible insects are very susceptible to contamination by pesticides and other chemicals [248]. Edible insects can thus accumulate harmful pesticide residues in their bodies, either for human use or as animal feed, which poses a significant safety challenge for edible insects. In contrast, edible insects raised under artificially managed indoor conditions can ensure their safety as food or feed. In terms of nutrient content, the nutritional content of field-harvested and farm-harvested insects of the same species is different [39]. For wild insects, the variety and distribution of nutrients is broad and balanced in quantity, which is related to their more complex food sources in the wild. For farmed insects, human intervention and the insect’s relatively homogeneous food source can result in particularly high levels of one of the nutrients of particular interest. This is because farmed insects are fed specially formulated foods. For example, fish oil or Perilla Seed is added [25,26].

From the point of view of health for humans and animals, farmed harvested insects seem to be healthier due to the lower safety issues associated with farmed insects. However, we cannot treat this issue in a one-sided manner. Some studies have reported that the high levels of mineral elements (such as iron and zinc) in certain wild insects can be beneficial for patients with micronutrient deficiencies [81,249]. The fatty acid composition and oil content of insects from wild-caught and farm-raised sources are also different [250]. Thus, both wild and farmed insects have their own pros and cons. For wild-harvested insects, we cannot control the environment and food sources, nor can we completely eliminate the potential safety issues. Conversely, for farmed insects, we can artificially alter the nutritional diversity of wild insects to better meet human needs for nutrient variety and content, as well as to better promote human health. Perhaps this purpose can be achieved by increasing the diversity of food sources for farmed insects. This brings us to the question of strategies to improve the nutrition of farmed insects.

### 4.3. Novel Strategies for Farming Insects

Killing insects from their natural environment is considered irrational, a destruction of natural resources, and has many limiting factors. For this reason, large-scale farming of edible insects is a reasonable and viable option [251]. The purpose of human insect farming is to make edible insects better able to meet human needs for nutrition and health. To this end, humans will develop and improve new strategies for farming insects whenever possible. Simply put, healthier and more productive edible insects are farmed by improving the environment in which they are farmed and by enriching their food sources [25,252]. Since the nutritional value of insects is mainly determined by the type of insect, the food source of the insect and the production and processing method, it is possible to develop highly nutritious cultured insects from these three perspectives [19].

First, insects raised on a large scale need to have obvious advantages, such as fast growth, abundant sources of feed, high reproductive capacity, high nutritional value, and adaptability [253,254]. In addition to this, the insects used for breeding and for food purposes must be safe and non-toxic for humans. Insects such as yellow mealworms, silkworms, palm weevils, and crickets are good species of insects that can be farmed on a large scale. Nowadays, insect species that can consume waste are more popular among humans. Examples include *Tenebrio molitor*, *Acheta domesticus*, and *Cockroaches* [255,256]. These insects can effectively transform various types of waste biomass, thus converting waste from the food production process into high-value insect nutrients. This in turn reduces the cost of insect production and waste of waste food [257]. As for the food source of insects, what must be considered is whether the supply can be sustained, the form of supply, whether it is safe and sanitary, and whether it meets the legal requirements [258,259]. Secondly, the nutritional factor has to be taken into account. This is because people breed insects to obtain highly nutritious food. Sometimes, insects can be supplemented with specific food components in order to enhance certain nutrients in their bodies to achieve the goal. For instance, giving a diet rich in linseed oil to three insects, house crickets (*Acheta domesticus*), lesser mealworms (*Alphitobius diaperinus*) and black soldier flies (*Hermetia illucens*), significantly increased the alpha-linolenic acid content in the insects. The results showed that diets supplemented with 4% linseed oil increased n-3 fatty acid content by 10–20 times in all three species [260]. The way insects are processed and produced is the last step that affects their nutritional value. Regardless of the method used to process insects, it is a basic requirement to ensure that the insects do not lose their nutrients and that the insects’ active ingredients are not inactivated. The influence of the processing method on the nutritional composition and biological activity of edible insects will be described below.

Today, the farming of edible insects has become a new industry in some regions, for example, the rapid development of insect farming in East Africa has led to some profitable businesses [261]. Crickets have also been factory farmed and processed in Asian and Western countries. Companies in North America and Europe are already marketing crickets as packaged food [262]. The development of technology has given new forms of insect farming. A recently published article analyzed the feasibility of using insect cells to produce insect products [263]. This is certainly a brand-new strategy for insect farming.

### 4.4. Sustainability of Edible Insects

Insects consumed by humans need to consider their sustainability in many aspects, such as ecological sustainability, sustainability of sources, sustainability of consumption, etc. [264]. Insect farming has advantages over traditional farming as a sustainable food source. For example, less demand for land and water; lower greenhouse gas emissions; high feed conversion efficiency and abundant food sources [265]. The sustainability of farmed insects for food and feed requires a life cycle assessment of them. In fact, choosing the right method for insect farming is not only beneficial to the environment, but can also lead to sustainable production [266]. Based on standardized life cycle assessment methods [267,268], life cycle assessment of the production and processing of cultured insects can be divided into three main stages. The first is the raw material needed to produce the insects. The second is the pretreatment of harvested insects, including harvesting, sterilization, drying, defatting, and grinding. The third is the additional processing used to produce food [266]. Although difficulties and challenges remain for the sustainability of the industrialization of edible insects, numerous studies and the large-scale farming and consumption of insects worldwide suggest that the industrialization of edible insects will further contribute to their sustainability and the expansion of production [269].

### 4.5. The Safety of Edible Insects

The market for edible insects is gradually expanding, which poses a great challenge to the quality control and safety of edible insects [39]. Potential safety issues for edible insects include allergic reactions, contamination with pathogenic microorganisms, pesticide residues, excessive levels of heavy metals, parasites and harmful toxins [270,271,272].

#### 4.5.1. Allergic Reactions

Insect allergy, like other food allergies, is the most serious safety concern for many insect eaters [271]. According to statistics, the World Health Organization has identified 239 possible allergens from arthropods, with grasshoppers and locusts accounting for the majority of these allergens [273]. The majority of insect allergens are proteins, such as hyaluronidase, phospholipase A, microtubulin, arginine kinase, and proto-myosin [271,273]. Breathing difficulties, asthma, redness, gastrointestinal issues, itching, tachycardia, hives, and, in severe situations, fainting are all indications of insect allergies [274]. According to some research, ingesting insects can cause allergic reactions comparable to those seen with seafood. For example, *Gryllus bimaculatus* and shrimp are cross-allergenic, presumably due to the presence of an allergen termed proto-myosin in both [275]. As a result, the exploitation of edible insects necessitates additional research on insect allergies, particularly the allergenicity of insect proteins and the potential for allergen transmission, in order to better safeguard insect food [276].

It’s comforting to know that allergens in edible insects can be processed in a variety of methods to lessen Allergenicity. Heat treatment is known to impact the allergenicity of proteins, but it does not necessarily eradicate the allergenicity of protein-based allergens entirely [277]. Furthermore, allergen sensitivity reduction via fermentation and hydrolysis appears to be quite beneficial [278]. Acidic and alkaline protease digestion and heat treatment, for example, were found to significantly reduce the allergenicity of protein-like substances in *Acheta domesticus*, *Schistocerca gregaria*, and *Tenebrio molitor*, with the desert locust *Schistocerca gregaria* exhibiting almost complete immunoreactivity after treatment [279]. In a study evaluating the allergenicity of *Acheta domesticus* crickets, it was discovered that proto-myosin in roasted insects was very stable and not easily hydrolyzed. Furthermore, *Acheta domesticus*’s cross-reactivity with crustaceans allows for allergic reactions comparable to those seen in fish and shrimp when *Acheta domesticus* proteins are consumed [280]. The researchers discovered that after treating the cricket *Gryllodes sigillatus* with alkaline protease and gastrointestinal protease, the protein hydrolysates obtained were not only highly active but that the hydrolysates at 60–85% hydrolysis were the least reactive towards proto-myosin [190]. According to the findings of the preceding investigations, adequate food processing methods are most likely to diminish the cross-reactivity and allergenicity of edible insects. As a result, whether employing insects as food or medication, proper processing processes should be used to eliminate allergenicity. Simultaneously, special consideration should be given to the fact that insects should be evaluated and studied in the manner in which they are employed and ingested. This will not only increase the safety of edible insect use but will also help the edible insect industry grow.

#### 4.5.2. Contamination by Pathogenic Microorganisms

Insects may also carry diseases that can be passed to people. The risk of zoonotic disease transmission through edible insects, on the other hand, is typically regarded as negligible [276]. This is because most insects consumed by humans and animals feed on plants and do not act as direct carriers of infections. Most insect-specific microorganisms do not pose a threat to humans [276,281].

But viruses are different from ordinary microorganisms, and unknowingly ingesting insects with viruses in excess may threaten human health. One study found that more than 70 virus species have been detected in edible insects, 36 of which can cause insect death or human disease [259]. At present, virus infection of edible insects cannot be completely eliminated, so preventive measures are the only useful method [259]. In addition, many potentially pathogenic human bacteria are present in insects. For example, Vibrio, Streptococcus, Staphylococcus, and Clostridium are present in edible insects sold in the EU [282]. Moreover, cases of microbial food-borne infections and poisoning caused by the consumption of insects have been reported [283].

#### 4.5.3. Pesticide Residues

For insects raised in captivity, pesticide residues are not a potential threat to insect safety. However, the opposite is true for food insects harvested in the wild. Because insects in the wild are not under human control, they can more easily ingest food sprayed with pesticides, which in turn leads to enrichment of pesticide residues in the insects. For example, pesticide residues and enrichment have been found in yellow mealworms, which are often consumed by humans [284,285]. Therefore, safe and controllable cultured edible insects can avoid the risk of pesticide residues to human health.

#### 4.5.4. Heavy Metal Content Exceeds the Standard

It is well known that the intake of foods containing excessive amounts of heavy metals can seriously affect the health of humans and animals [286]. The accumulation of heavy metals in edible insects is also a potential safety concern for field-harvested insects. The levels of heavy metals in insects are related to the insect species, growth stage, growth environment and food source [287,288]. There are reports of high levels of lead in local food grasshoppers in Mexico due to the fact that these grasshoppers forage in highly contaminated mines [289]. Heavy metals in edible insects originate from both habitat environment and human pollution. The most common heavy metals reported in edible insects are cadmium, lead, arsenic and mercury [9]. Likewise, farming insects will greatly reduce the safety risks associated with heavy metals.

#### 4.5.5. Other Security Concerns

In addition to the safety concerns mentioned above, the presence of parasites in edible insects also needs to be considered. Some parasites are likely to be transmitted to humans through the consumption of insects. Examples include *Dicrocoelium dendriticum*, *Entamoeba histolytica*, *Giardia lamblia* and *Toxoplasma* [290]. In addition, some harmful toxins and Antinutrients in insects can also threaten human health [291,292].

### 4.6. Processing of Edible Insects

Traditionally, insects have been processed in simple ways to improve their taste and eating quality, such as steaming, roasting, smoking, frying, and stewing. With the rise of new processing technologies, the processing of edible insects has become more elaborate [293].

The nutritional composition of insects can vary depending on the method of their acquisition and processing conditions. In the case of insect protein acquisition, conventional extraction methods are acidic or alkaline extraction, organic solvents or isoelectric point precipitation. However, these methods affect the purity and stability of the target to a greater or lesser extent and can produce waste that is hazardous to the environment [294,295]. Toxic solvents, for example [296]. Therefore, the processing and extraction of the target from edible insects needs to be linked to the concept of biorefinement. Nowadays, some novel and environmentally friendly extraction methods are gradually chosen by researchers due to their significant advantages [293]. For example, ultrasound-assisted extraction, supercritical fluid extraction, pressurized liquid extraction and microwave-assisted extraction. It was shown that the protein yield could be significantly improved by using ultrasound treatment of defatted yellow flour samples [297]. The protein content was increased by 28% after 15 min of sonication. Supercritical fluid extraction has shown good application in the extraction of insect oil. Supercritical CO_2_ extraction of insect oil can achieve the same extraction rate as the conventional method, but this extraction method is safer and more environmentally friendly [298]. Pressurized liquid extraction has also been used to extract insect components, with the significant advantage of reducing extraction time and not producing harmful substances [299,300]. Besides, microwave-assisted extraction has shown good results in the enzymatic digestion of insect proteins, which can assist the enzymatic digestion and extraction process [301]. When processing and extracting insect components, a single method is often not as effective as it could be, and more often than not, several methods need to be used simultaneously to achieve the processing goals.

It is also important to note that some insects should be limited or reduced in their application to food due to the presence of excessive nutrients. The health effects of insects on humans may not be what most people think they are. With the increased demand for healthier diets, foods high in cholesterol that can cause human disease have been restricted for use in foods [302]. Therefore, some insect species with high saturated fat content are not popular when used as food [250]. For example, in termites and palm weevils, there are 28.2 and 31.8 g of fat per 100 g of dry weight, and 13,900 mg and 17,500 mg of saturated fat [39]. Therefore, the refining of insect oil may facilitate the use of high cholesterol insects in food products. In analogy to other foods, cholesterol in insect oil may be removed by β-cyclodextrin. It has been found that the addition of 1.5% (*w*/*w*) of β-cyclodextrin to milk can remove up to 99.4% of cholesterol [303].

On the other hand, the method of processing and obtaining insect active ingredients can also affect their functional activity. The method of extraction for proteins is the primary influencing factor directly altering the function of insect proteins [304]. Alkali extraction, water extraction, dry fractionation, ultrasonic extraction, and ultra-high pressure extraction are now used to extract insect active proteins [293]. A study on the extraction of proteins from three different edible insect larvae (*Tenebrio molitor*, *Allomyrina dichotoma*, and *Protaetia brevitarsis*) discovered that the technical functioning of the insect proteins could be successfully improved by altering the extraction conditions [305]. Extraction method selection and optimization can not only increase the extraction rate of insect proteins but also reduce protein activity damage [306]. Furthermore, the presence of the exoskeleton can decrease the digestion of insect proteins [307]. The predominant component of these exoskeletons, however, is generally chitin, which can be removed through processing or conversion through eating [41,54]. It has been demonstrated that insects with their exoskeletons removed have up to 98% protein digestibility [308]. As a result, whether employing insect proteins as food additives or producing medications, careful consideration should be given to the process of acquiring and purifying them. Unlike proteins, the extraction process does not affect the content of fatty acids; nevertheless, the kind of fat and the rate of fat extraction can vary depending on the extraction method [11]. A study of four insect species using two industrial extraction methods, aqueous and Soxhlet extraction, and a laboratory method (Folch extraction) showed that the Folch extraction method yielded the most fatty ω-6 fatty acids and the highest extraction rates, while the aqueous extraction method yielded the highest levels of health-related ω-3 fatty acids, although at a lower extraction rate [309]. This suggests that the extraction method can directly influence the characteristics or type of insect fat and that special attention needs to be paid to the choice of method used to obtain insect fat. In addition to proteins and fats, chitin and chitosan are also obtained with environmental considerations in mind [310]. The extraction of chitin from insects is considered to be more advantageous than that from marine crustaceans. Traditional extraction methods include demineralization and deproteinization [310]. Today, greener extraction methods are being developed, for example, using microbial extraction [311]. Extraction using natural deep eutectic solvents [312], and improved acid-base methods [313] can obtain greener and better quality chitin and chitosan from insects.

### 4.7. Purity and Stability of Insect Extract Components

When people study a certain ingredient, what they must consider is the purity and stability of that ingredient. Similar to common food ingredients, in the process of extracting insect ingredients, the purity and stability of the extract ingredients can be improved by optimizing the extraction method and taking protective measures [314,315]. For example, the oxidative stability of insect oils can be improved when obtained using ethanol-isopropanol as a solvent and under sonication, and the insect oils obtained by this method contain lower levels of peroxides, conjugated dienes and trienes, and free fatty acids [314]. The insect oil obtained using pressurized n-propane extraction also has good oxidative stability, and this method also has the advantage of being less time-consuming [315]. As mentioned earlier, when choosing a suitable extraction method, not only can the extraction efficiency be improved, but also higher yields and more stable products can be obtained.

In addition to these considerations, the economic and environmental issues linked with insect consumption must be considered, as they cannot be consumed at the expense of economic development and environmental damage [294].

## 5. Edible Insects and Human Life

To date, the world’s population is still growing and the demand for food is still increasing, and people are having to work harder to find new sources of food. The edible insect industry is gradually coming into people’s lives. Today, edible insects are widely used and can be found in many kinds of food products.

To begin with, adding insect meals to flour can increase the nutritional and organoleptic aspects of the product. The addition of grasshopper flour and defatted grasshopper flour to bread, for example, can change the rheological properties of the bread, making it softer, and can also improve the nutritional value of the bread, with 200 g/kg of grasshopper flour added to a bread recipe increasing the protein content by up to 60% [316]. According to studies, adding 10% cricket powder to wheat flour increases bread popularity, and bread containing cricket powder has a greater nutritional value in terms of fatty acid composition, protein content, and essential amino acid content [317]. When utilizing cricket powder, the safety concerns related to the microorganisms carried by crickets are also underlined. The inclusion of cricket powder in the creation of gluten-free bread results in gluten-free bread with outstanding process qualities and high protein content, as well as strong antioxidant activity [318,319]. Furthermore, adding silk flours to soba noodles boosts the protein content of the noodles while decreasing the recommended cooking time. Most significantly, it conceals the taste of soba, which most people dislike [320].

Similarly, adding insects to meat products can be advantageous. For example, adding edible silkworm pupae to the beef batter can increase its physicochemical qualities such as pH, viscosity, hardness, and chewiness. These effects are comparable to adding transglutaminase to beef batter [321]. Furthermore, adding cricket powder to emulsified meat products boosts the protein and trace elements in the meat emulsion [322]. Mealworm larvae and silkworm pupae can both be used as a source of protein in emulsified sausages [323]. Also, insects are frequently incorporated into a variety of snack meals, including biscuits, chocolate, tortilla-style chips, and other munchies [293,324].

Edible insects are also employed in fermented foods. Studies on insect fermentation treatments have revealed that the secondary metabolites created by fermentation are more nutritious, as well as having antibacterial and medicinal properties [325]. Edible insect fermented products can be used to create a variety of food products such as pastes, powders, sauces, and fermented dishes incorporating insects [325]. Pupae peptides have been researched for their effect on the quality of fermented dairy products, and the results suggest that pupae peptides greatly improve the acidity and textural aspects of yogurt [326]. Another study used yellow meal worm larvae in a soy sauce fermentation procedure to make liquid fermented sauces. The quantity of free amino acids and amino acid derivatives was observed to be increased in these fermented condiments during the experiments. There were a lot of glutamic acids, alanine, aspartic acid, serine, isoleucine, lysine, phenylalanine, and valine [327].

**Figure 9:**
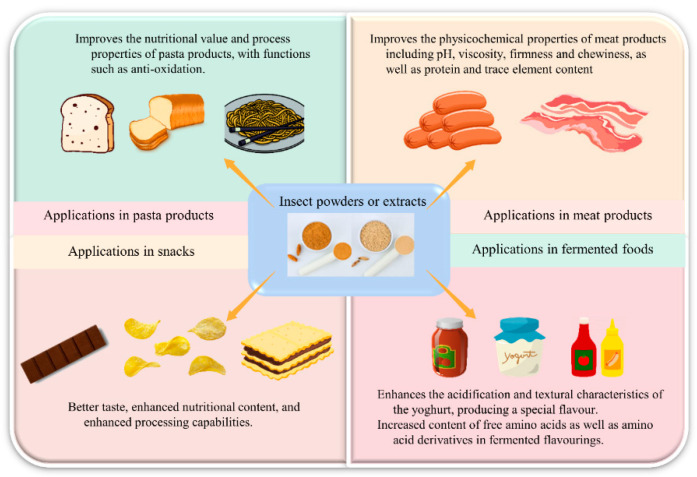
Edible insects in food applications.

There are several new packaged foods containing insects on the market today, as well as companies creating them, but they are not yet mainstream foods in terms of sales [293]. Insect-based foods are highly healthy and sustainable, and they may sell better if legal regulatory restrictions can be addressed [328]. Figure 9 indicated the usage of edible insects in food products.

A broad variety of edible insects are utilized in medications and health goods, in addition to food. In China, insects and their derivatives are employed directly or indirectly to cure a wide range of ailments [30]. In Chinese medicine, insects have been employed to heal ailments for over 2000 years [14]. The previous section’s in vivo and in vitro investigations established the existence of therapeutic qualities in edible insects. Insect therapy is increasingly using edible insects [31]. Maggots, for example, can treat wounds by lowering infection and increasing healing; blood-sucking insects like horseflies can treat mending blood issues; and whole-body extracts of insects like bees, moths, and cockroaches can be utilized as anti-cancer and anti-bacterial medications [12,13].

Simple direct consumption does not meet people’s needs for nutrition and safety of food. In the future, the applications of edible insects in human life will be more diverse. In order to better consume edible insects, new food processing technologies should be applied more to obtain and analyze edible insect ingredients, so that more new products can be developed.

## 6. Current Legislation on Edible Insects

Edible insects are high in nutrients such as fat, protein, and minerals and can be used as an alternative food source. It is therefore essential to promote the legal recognition of edible insects as a source of food and functional active ingredients. The range of applications and potential of the insect food industry has been largely overlooked by legislators [329]. Previously, insects were too small-scale and not widely enough used as food to be included in the scope of the legislation. The lack of clear legislation on the rearing, consumption, and commercialization of edible insects in most countries has severely hampered the development of edible insects and their potential to benefit human health [330]. An article from 2021 compares the laws around the world governing the use of insects as food and feed. The laws governing edible insects in the European Union, the United States, Canada, Australia, China, Japan, and other nations were contrasted and examined in the article. The findings show that each nation has its own legislation, despite the fact that all governments place a high importance on the safety of edible insects. It is challenging to distribute and consume edible insects internationally due to the diversity of restrictions in different nations [17]. Regulations on edible insects are gradually being developed as the availability of insects for rearing, eating, and producing goods grows. The European Commission has acknowledged that using insects in feed and food can give significant environmental, economic, and food security benefits. In 2013, a book entitled “Edible Insects: Future Prospects for Food and Feed Security”, published by the Food and Agriculture Organization of the United Nations (FAO), highlighted for the first time the lack of a legal framework as a major obstacle limiting the adoption of insects as food and feed in Europe [3].

Many governments are currently taking the first steps toward regulating particular insects as food sources. Some nations, including Belgium, the United Kingdom, the Netherlands, the Kingdom of Denmark, Finland, and Kenya, have rules governing the legality of certain cricket species [331,332,333]. However, the legislation on edible insects in these countries is not clear and complete. Since the use of insects as food in several European countries, The European Food Safety Authority (EFSA) and the Federal Agency for Safety of the Food Chain (FASFC) have started specific regulations on the consumption of insects by humans and animals. These regulations are concerned with the microbiological standards that apply to edible insects as well as the status of insects as novel foods. However, the majority of these laws are based on animal diet and feed regulations. In Europe, edible insects were initially recognized as the food of animal origin [334]. Before 2015, insect regulations mostly treated insects as contaminants in food, and there were no regulations that explicitly mentioned insects as food sources [335]. According to EFSA, four new insect foods have been legally regulated, including the lesser mealworm (*Alphitobius diaperinus* larva) [47], whole house crickets (*Acheta domesticus*) [336], migratory locust (*Locusta migratoria*) [46], defatted house cricket (*Acheta domesticus*) powder [337]. The above four new insect food products are regulated by Regulation (EU) 2015/2283 and have been evaluated for safety by EFSA. Before this, EU Regulation 2017/893, which came into force on 1 July 2017, already allowed the use of seven insect species as aquaculture feed [338]. The EFSA also carried out allergenic risk assessments and analytical tests on insects used as food and feed [338].

A variety of reasons influence the development of edible insect regulations. The first consideration is food safety, which must always come first. Following that are consumer acceptance and environmental preservation. At the same time, the sustainability of the insect source must be considered. Insect legislation also necessitates government engagement, taking into mind existing regulatory systems [339]. Of the many factors, governmental factors are the most critical, and regulations in different countries in different places can directly affect the development of various insect businesses [340]. And consumer acceptance can directly influence the scale of insect consumption. There is still an aversion to insects in the Western world. Therefore it is essential to turn insects into a product that will appeal to people [341]. The multifaceted elements of insects as food must be fully considered in the establishment of future regulations [333].

## 7. Conclusions and Perspectives

This essay examines the biological functions and nutritional worth of edible insects, as well as their application and safety in food and medicine. Edible insects are a fantastic source of supplemental nutrients for the human body since they are high in protein, fat, vitamins, and minerals. The amino acid and some trace element need of the human body can be satisfied by these edible insects. Additionally, because they contain a variety of functional active components, edible insects have the potential to be exploited in the development of particular medications for the diagnosis, treatment, and prevention of human diseases. Even though medicinal insects have been used for a very long time in traditional medicine, modern biomedicine is slowly revealing how they work.

Despite the practicality of using insects as food and medicine, achieving human health still faces numerous difficulties. It is necessary to describe in greater detail the therapeutic benefits and modes of action of edible insects as well as their active components. To determine whether the various functional effects have similar impacts on humans, experiments to explore the biological functions of edible insects must also be conducted in as many human experiments as possible. To better reduce the allergenicity of edible insects, the future study must concentrate on the cross-reactivity and allergenicity of edible insects as well as the applicability of processing techniques for various species of insects.

To better apply edible insects to daily eating in the future, several obstacles must be resolved. First and foremost, as a foundation for their development and use, edible insect use as food or medicine needs to be supported by international and national laws and policies. Second, researchers should focus more on undiscovered food insect species because there are probably many undiscovered edible bug species and those with therapeutic characteristics. In addition, the nutritional value of edible insects should be standardized, and the selection and compounding of different insects may lead to insect products with higher nutritional value and better therapeutic effects. In addition, special attention should be paid to the safety and stability of edible insects, and the development of non-toxic and safe insect products is the goal we are pursuing [54]. Overall, edible insects have a significant role to play in sustaining human health and supplying nourishment, and they could represent the future of both food research and the food industry.

## Figures and Tables

**Table 1:** Proximate composition of insect matter.

Scientific Name	Moisture	Protein	Fat	Ash	Fiber	Reference
*Antheraea pernyi*	7.6 ^a^	71.9 ^a^	20.1 ^a^	4 ^a^	NA	[34]
*Clanis bilineata tsingtauica*	NA	65.5 ^a^	23.68 ^a^	2.17 ^a^	3.77 ^a^	[35]
*Oxya chinensis*	NA	20.8 ^a^	2.2 ^a^	1.2 ^a^	1.2 ^a^	[35]
*Schistocerca piceifrons piceifrons*	NA	80.26 ^a^	6.21 ^a^	3.25 ^a^	12.56 ^a^	[36]
*Gryllus bimaculatus*	NA	58.32 ^a^	11.88 ^a^	9.69 ^a^	9.53 ^a^	[37]
*Gonimbrasia belina*	5.68 ^a^	46.7 ^a^	14.04 ^a^	11.38 ^a^	NA	[38,39]
*Hermetia illucens*	5.76 ^a^	34.9 ^a^	27.93 ^a^	7.5 ^a^	NA	[38]
*Macrotermes subhylanus*	6.40 ^a^	52.74 ^a^	6.36 ^a^	6.41 ^a^	NA	[38]
*Macrotermes bellicosus*	2.82 ^a^	20.4 ^a^	NA	2.9 ^a^	2.7 ^a^	[40]
*Macrotermes notalensis*	2.98 ^a^	22.1 ^a^	NA	1.9 ^a^	2.2 ^a^	[40]
*Brachytrypes spp.*	3.41 ^a^	6.25 ^a^	NA	1.82 ^a^	1.01 ^a^	[40]
*Cytacanthacris aeruginosus unicolor*	2.56 ^a^	12.1 ^a^	NA	2.1 ^a^	1.5 ^a^	[40]
*Zonocerus variegatus*	2.61 ^a^	26.8 ^a^	NA	1.2 ^a^	2.4 ^a^	[40]
*Analeptes trifasciata*	2.19 ^a^	29.62 ^a^	NA	4.21 ^a^	1.96 ^a^	[40]
*Anaphe infracta*	2.73 ^a^	20 ^a^	NA	1.6 ^a^	2.4 ^a^	[40]
*Anaphe recticulata*	3.21 ^a^	23 ^a^	NA	2.5 ^a^	3.1 ^a^	[40]
*Anaphe spp.*	2.52 ^a^	18.9 ^a^	NA	4.1 ^a^	1.68 ^a^	[40]
*Anaphe venata*	3.34 ^a^	25.7 ^a^	NA	3.2 ^a^	2.3 ^a^	[40]
*Cirina forda*	4.40 ^a^	20.2 ^a^	NA	1.5 ^a^	1.8 ^a^	[40,41]
*Apis mellifera*	3.82 ^a^	21 ^a^	14.5 ^a^	2.2 ^a^	2 ^a^	[39,40,42]
*Analeptes trifasciata*	2.65 ^a^	20.1 ^a^	NA	1.5 ^a^	3.3 ^a^	[40]
*Oryctes boas*	1.91 ^a^	26 ^a^	NA	1.5 ^a^	3.4 ^a^	[40]
*Rhynchophorus phoenicis*	2.74 ^a^	28.42 ^a^	NA	2.7 ^a^	2.82 ^a^	[40,41]
*Gynanisa maja*	9.2 ^a^	55.92 ^a^	12.1 ^a^	7.4 ^a^	NA	[43]
*Macrotermes falciger*	4.1 ^a^	43.26 ^a^	43.0 ^a^	7.3 ^a^	NA	[43]
*Ruspolia differens*	NA	44.3 ^a^	46.2 ^a^	2.6 ^a^	4.9 ^a^	[41,43]
*Imbrasia belina*	NA	56.8 ^a^	12.9 ^a^	10.4 ^a^	NA	[44]
*Gryllodes sigillatus*	NA	70 ^a^	18.23 ^a^	4.74 ^a^	3.65 ^a^	[45]
*Schidtocerca gregaria*	NA	76 ^a^	12.97 ^a^	3.33 ^a^	2.53 ^a^	[45]
*Locusta migratoria*	4.2 ^a^	48.7 ^a^	38.1 ^a^	2.3 ^a^	8.8 ^a^	[46]
*Alphitobius diaperinus*	2.74 ^a^	58.76 ^a^	25.9 ^a^	3.5 ^a^	6.08 ^a^	[47]
*Rhynchophorus ferrugineus*	67.9 ^b^	18.0 ^a^	58.8 ^a^	2.4 ^a^	NA	[48]
*Hermetia illucens*	61.2 ^b^	17.5 ^b^	14 ^b^	NA	6.8 ^b^	[39,49]
*Chilecomadia moorei*	60.2 ^b^	15.5 ^b^	29.4 ^b^	NA	4 ^b^	[49]
*Blatta lateralis*	69.1 ^b^	19 ^b^	10 ^b^	NA	5 ^b^	[49]
*Musca domestica*	74.8 ^b^	19.7 ^b^	1.9 ^b^	NA	6.5 ^b^	[49]
*Zophobas morio*	57.90 ^b^	19.70 ^b^	17.70 ^b^	1.00 ^b^	6.60 ^b^	[33,50]
*Tenebrio molitor*	61.00 ^b^	18.40 ^b^	16.80 ^b^	1.20 ^b^	5.40 ^b^	[33,50,51]
*Galleria mellonela*	58.50 ^b^	14.10 ^b^	24.90 ^b^	0.60 ^b^	12.50 ^b^	[33,50]
*Bombyx mori*	82.70 ^b^	9.30 ^b^	1.40 ^b^	1.10 ^b^	2.20 ^b^	[33,41]
*Acheta domesticus*	69.20 ^b^	20.50 ^b^	6.80 ^b^	1.10 ^b^	10.00 ^b^	[33,41,50]
*Oecyphylla smaragdina*	59.50 ^b^	10.80 ^b^	10.80 ^b^	NA	NA	[39]

^a^: % dry weight, ^b^: g/100 g fresh weight. NA: not available.

**Table 2:** Amino acid composition of insects.

Scientific Name	Ile	Leu	Lys	Met	Cys	Phe	Tyr	Thr	Trp	Val	Arg	His	Ala	Asp	Glu	Gly	Pro	Ser	Reference
*Antheraea pernyi*	79.5 ^a^	32.4 ^a^	45.4 ^a^	14.7 ^a^	1.5 ^a^	81 ^a^	20.6 ^a^	46.4 ^a^	40.5 ^a^	66.3 ^a^	41.2 ^a^	29.4 ^a^	62.6 ^a^	64.1 ^a^	127.4 ^a^	44.2 ^a^	122.2 ^a^	46.4 ^a^	[34]
*Bombyx mori*	57 ^a^	83 ^a^	75 ^a^	46 ^a^	14 ^a^	51 ^a^	54 ^a^	54 ^a^	6 ^a^	56 ^a^	68 ^a^	25 ^a^	55 ^a^	109 ^a^	149 ^a^	46 ^a^	40 ^a^	47 ^a^	[34]
*Gryllodes sigillatus*	25.6	57.8	38.4	15.9	11.1	22.0	31.8	36.8	NA	47.0	46.6	17.2	58.0	72.8	106.6	40.7	54.2	40.4	[45]
*Schistocerca gregaria*	28.2	77.7	35.1	8.2	3.6	18.7	33.1	35.5	NA	56.6	39.8	20.6	88.8	66.1	107.5	49.4	67.1	33.7	[45]
*Hermetia illucens*	7.62 ^b^	12.1 ^b^	11.9 ^b^	3.37 ^b^	1.02 ^b^	7.56 ^b^	12.1 ^b^	6.82 ^b^	3.00 ^b^	12.9 ^b^	12.3 ^b^	5.94 ^b^	12.2 ^b^	16.5 ^b^	19.7 ^b^	9.14 ^b^	10.2 ^b^	7.02 ^b^	[49]
*Chilecomadia moorei*	6.51 ^b^	10.1 ^b^	8.72 ^b^	2.49 ^b^	0.87 ^b^	5.47 ^b^	7.95 ^b^	5.74 ^b^	1.56 ^b^	9.71 ^b^	11.7 ^b^	4.08 ^b^	8.67 ^b^	12.9 ^b^	16.4 ^b^	6.53 ^b^	9.52 ^b^	7.88 ^b^	[49]
*Blatta lateralis*	7.73 ^b^	12 ^b^	12.8 ^b^	3.35 ^b^	1.44 ^b^	7.67 ^b^	14.3 ^b^	7.89 ^b^	1.66 ^b^	12.3 ^b^	14 ^b^	5.49 ^b^	16.7 ^b^	15.1 ^b^	22.6 ^b^	12.4 ^b^	10.6 ^b^	8.38 ^b^	[49]
*Musca domestica*	8.1 ^b^	12.4 ^b^	12.6 ^b^	5.84 ^b^	1.4 ^b^	7.91 ^b^	9.26 ^b^	7.54 ^b^	2.4 ^b^	11 ^b^	12.1 ^b^	5.71 ^b^	11.7 ^b^	16.3 ^b^	21.1 ^b^	8.43 ^b^	8.36 ^b^	6.97 ^b^	[49]
*Zophobas morio*	9.3 ^b^	19.1 ^b^	10.3 ^b^	2.1 ^b^	1.5 ^b^	6.8 ^b^	13.7 ^b^	7.8 ^b^	1.8 ^b^	10.3 ^b^	9.6 ^b^	6.0 ^b^	14.3 ^b^	15.8 ^b^	24.2 ^b^	9.5 ^b^	10.8 ^b^	9.2 ^b^	[33,50]
*Tenebrio molitor*	8.6 ^b^	14.3 ^b^	11.2 ^b^	2.6 ^b^	1.5 ^b^	7.5 ^b^	14.3 ^b^	6.4 ^b^	1.7 ^b^	12.2 ^b^	10.3 ^b^	6.5 ^b^	13.7 ^b^	16.2 ^b^	22.8 ^b^	9.9 ^b^	12.1 ^b^	9.1 ^b^	[33,50,57]
*Galleria mellonella*	6.3 ^b^	12.4 ^b^	7.9 ^b^	2.2 ^b^	1.1 ^b^	5.3 ^b^	8.8 ^b^	5.9 ^b^	1.2 ^b^	6.8 ^b^	7.1 ^b^	3.3 ^b^	9.4 ^b^	13.4 ^b^	19.5 ^b^	7.4 ^b^	9.5 ^b^	10.5 ^b^	[33,50]
*Acheta domesticus*	9.4 ^b^	20.5 ^b^	11.0 ^b^	3.0 ^b^	1.7 ^b^	6.5 ^b^	10.0 ^b^	7.4 ^b^	1.3 ^b^	10.7 ^b^	12.5 ^b^	4.8 ^b^	18.0 ^b^	17.2 ^b^	21.5 ^b^	10.4 ^b^	11.5 ^b^	10.2 ^b^	[33,50,58]
*Gryllus bimaculatus*	9.2 ^b^	16.5 ^b^	11.4 ^b^	3.5 ^b^	1.6 ^b^	7.4 ^b^	11.7 ^b^	8.1 ^b^	2.2 ^b^	13.6 ^b^	11.4 ^b^	5.2 ^b^	19.3 ^b^	19.7 ^b^	24.4 ^b^	12.4 ^b^	12.5 ^b^	10.5 ^b^	[58]
*Gonimbrasia belina*	13.0 ^c^	18.3 ^c^	25.6 ^c^	4.1 ^c^	1.1 ^c^	13.5 ^c^	22.3 ^c^	18.4 ^c^	4.8 ^c^	19.1 ^c^	45.7 ^c^	18.4 ^c^	23.6 ^c^	31.3 ^c^	43.5 ^c^	17.9 ^c^	18.6 ^c^	17.5 ^c^	[43]
*Gynanisa maja*	18.8 ^c^	27.2 ^c^	40.2 ^c^	8.2 ^c^	2.2 ^c^	19.8 ^c^	41.7 ^c^	22.6 ^c^	7.5 ^c^	20.9 ^c^	31.4 ^c^	25.3 ^c^	25.5 ^c^	39.9 ^c^	52.4 ^c^	19.9 ^c^	25.0 ^c^	23.1 ^c^	[43]
*Ruspolia differens*	26.1 ^c^	26.7 ^c^	57.4 ^c^	4.3 ^c^	0.7 ^c^	26.1 ^c^	25.3 ^c^	28.6 ^c^	0.3 ^c^	16.4 ^c^	49.8 ^c^	44.1 ^c^	26.6 ^c^	49.0 ^c^	84.3 ^c^	26.0 ^c^	19.0 ^c^	25.9 ^c^	[43]
*Macrotermes falciger*	18.9 ^c^	31.6 ^c^	37.2 ^c^	8.2 ^c^	1.3 ^c^	19.7 ^c^	34.4 ^c^	19.5 ^c^	3.5 ^c^	21.7 ^c^	30.1 ^c^	26.5 ^c^	27.4 ^c^	37.3 ^c^	46.8 ^c^	18.9 ^c^	19.3 ^c^	20.8 ^c^	[43]
*Imbrasia belina*	22.0 ^c^	35.0 ^c^	36.0 ^c^	9.0 ^c^	NA	25.0 ^c^	36.0 ^c^	27.0 ^c^	7.0 ^c^	NA	32.0 ^c^	17.0 ^c^	NA	NA	NA	NA	NA	NA	[44]
*Apis mellifera*	16.0 ^c^	25.0 ^c^	19.0 ^c^	NA	3.0 ^c^	2.0 ^c^	15.0 ^c^	16.0 ^c^	NA	17.0 ^c^	16.0 ^c^	7.0 ^c^	16.0 ^c^	26.0 ^c^	50.0 ^c^	14.0 ^c^	NA	14.0 ^c^	[42]
*Rhynchophorus ferrugineus*	8 ^c^	12 ^c^	11 ^c^	2 ^c^	1 ^c^	7 ^c^	21 ^c^	8 ^c^	1 ^c^	10 ^c^	10 ^c^	4 ^c^	11 ^c^	16 ^c^	25 ^c^	9 ^c^	10 ^c^	9 ^c^	[48]

^a^: g/kg protein, ^b^: g/kg fresh weight, ^c^: g/kg dry weight. Column heading abbreviations are as follows: Ile—isoleucine, Leu—leucine, Lys—lysine, Met—methionine, Cys—cysteine, Phe—phenylalanine, Tyr—tyrosine, Thr—threonine, Trp—tryptophan, Val—valine, Arg—arginine, His—histidine, Ala—alanine, Asp—aspartic acid, Glu—glutamic acid, Gly—glycine, Pro—proline, Ser—serine. NA: not available.

**Table 3:** Fatty acid composition of insect fats.

Scientific Name	C_10:0_	C_12:0_	C_14:0_	C_15:0_	C_16:0_	C_17:0_	C_18:0_	C_20:0_	SFA	C_14:1_	C_16:1_	C_17:1_	C_18:1_	C_20:1_	MUFA	C_18:2_	C_18:3_	PUFA	Reference
*Zophobas morio*	NA	<0.2 ^a^	1.7 ^a^	0.4 ^a^	52.8 ^a^	0.7 ^a^	12.6 ^a^	0.4 ^a^	68.6 ^a^	NA	0.7 ^a^	0.6 ^a^	66.0 ^a^	NA	67.3 ^a^	32.9 ^a^	1.1 ^a^	34.0 ^a^	[33,50]
*Tenebrio molitor*	NA	0.6 ^a^	5.2 ^a^	0.2 ^a^	25.5 ^a^	0.2 ^a^	4.0 ^a^	0.2 ^a^	35.9 ^a^	NA	4.8 ^a^	0.2 ^a^	66.4 ^a^	NA	71.4 ^a^	49.0 ^a^	2.2 ^a^	51.2 ^a^	[33,50]
*Galleria mellonella*	NA	<0.2 ^a^	0.4 ^a^	<0.2 ^a^	79.6 ^a^	<0.2 ^a^	3.4 ^a^	0.3 ^a^	83.7 ^a^	NA	5.1 ^a^	0.3 ^a^	124.0 ^a^	NA	129.4 ^a^	15.2 ^a^	1.1 ^a^	16.3 ^a^	[33,50]
*Bombyx mori*	NA	<0.2 ^a^	<0.3 ^a^	<0.4 ^a^	1.7 ^a^	<0.2 ^a^	1.2 ^a^	0.1 ^a^	3.0 ^a^	NA	0.1 ^a^	<0.2 ^a^	3.2 ^a^	NA	3.3 ^a^	3.5 ^a^	1.4 ^a^	4.9 ^a^	[33]
*Acheta domesticus*	NA	<0.2 ^a^	0.4 ^a^	<0.2 ^a^	15.6 ^a^	0.2 ^a^	5.8 ^a^	0.4 ^a^	22.4 ^a^	NA	0.9 ^a^	<0.1 ^a^	15.4 ^a^	NA	16.3 ^a^	22.9 ^a^	0.6 ^a^	23.5 ^a^	[33,50]
*Hermetia illucens*	0.69 ^a^	52.1 ^a^	12.0 ^a^	0.12 ^a^	16.1 ^a^	0.20 ^a^	2.45 ^a^	0.16 ^a^	83.82 ^a^	0.12 ^a^	4.96 ^a^	<0.08 ^a^	15.6 ^a^	<0.08 ^a^	20.68 ^a^	16.9 ^b^	0.65 ^a^	17.55 ^a^	[49]
*Chilecomadia moorei*	<0.10 ^a^	0.93 ^a^	0.95 ^a^	<0.10 ^a^	69.3 ^a^	<0.10 ^a^	2.19 ^a^	0.24 ^a^	73.61 ^a^	<0.10 ^a^	14.7 ^a^	<0.10 ^a^	149.0 ^a^	0.19 ^a^	163.89 ^a^	6.99 ^a^	0.45 ^a^	7.44 ^a^	[49]
*Blatta lateralis*	<0.20 ^a^	<0.20 ^a^	0.48 ^a^	<0.20 ^a^	17.4 ^a^	<0.20 ^a^	4.22 ^a^	<0.20 ^a^	22.1 ^a^	<0.20 ^a^	1.21 ^a^	<0.20 ^a^	40.9 ^a^	<0.25 ^a^	42.11 ^a^	21.6 ^a^	0.71 ^a^	22.31 ^a^	[49]
*Musca domestica*	<0.01 ^a^	0.02 ^a^	0.32 ^a^	0.17 ^a^	3.72 ^a^	0.10 ^a^	0.40 ^a^	0.04 ^a^	4.77 ^a^	0.02 ^a^	1.96 ^a^	<0.01 ^a^	2.89 ^a^	0.01 ^a^	4.97 ^a^	4.15 ^a^	0.45 ^a^	4.6 ^a^	[49]
*Imbrasia belina*	NA	<0.1 ^b^	<0.1 ^b^	NA ^b^	3.0 ^b^	NA ^b^	1.7 ^b^	<0.1^b^	4.9 ^b^	NA	0.1 ^b^	NA	1.8 ^b^	NA	1.7 ^b^	1.6 ^b^	3.7 ^b^	5.4 ^b^	[44]
*Ruspolia differens*	NA	NA	NA	NA	32.1 ^c^	NA	5.9 ^c^	NA	39.1 ^c^	NA	1.4 ^c^	NA	24.9 ^c^	NA	26.3 ^c^	29.5 ^c^	4.2 ^c^	33.8 ^c^	[41]
*Gryllodes sigillatus*	NA	0.10 ^c^	1.65 ^c^	0.24 ^c^	23.5 ^c^	0.32 ^c^	7.35 ^c^	0.40 ^c^	33.74 ^c^	0.09 ^c^	3.78 ^c^	0.29 ^c^	29.14 ^c^	1.03 ^c^	34.33 ^c^	29.78 ^c^	2.13 ^c^	31.91 ^c^	[45]
*Schistocerca gregaria*	0.07 ^c^	0.23 ^c^	1.68 ^c^	0.09 ^c^	23.26 ^c^	0.24 ^c^	9.27 ^c^	0.40 ^c^	35.3 ^c^	NA	1.80 ^c^	0.20 ^c^	36.22 ^c^	0.14 ^c^	38.35 ^c^	14.04 ^c^	11.35 ^c^	26.28 ^c^	[45]
*Polyrhachis vicina*	NA	0.7 ^c^	0.6 ^c^	0.1 ^c^	17.5 ^c^	0.2 ^c^	4.3 ^c^	0.3 ^c^	23.9 ^c^	NA	8.2 ^c^	0.4 ^c^	63.0 ^c^	0.7 ^c^	72.4 ^c^	2.1 ^c^	0.2 ^c^	2.5 ^c^	[66]
*Oecophylla smaragdina*	NA	0.9 ^c^	2.1 ^c^	0.2 ^c^	20.8 ^c^	0.3 ^c^	5.8 ^c^	1.0 ^c^	31.9 ^c^	NA	4.3 ^c^	0.5 ^c^	52.1 ^c^	1.6 ^c^	58.7 ^c^	7.0 ^c^	1.0 ^c^	8.4 ^c^	[66]
*Apis mellifera*	NA	0.3 ^c^	2.4 ^c^	NA	37.3 ^c^	NA	11.8 ^c^	NA	51.8 ^c^	NA	0.7 ^c^	NA	47.5 ^c^	NA	48.2 ^c^	NA	NA	NA	[42]
*Rhynchophorus ferrugi-neus*	NA	1 ^c^	1.6 ^c^	NA	49.4 ^c^	NA	0.1 ^c^	NA	53 ^c^	NA	NA	NA	46.9 ^c^	NA	46.9 ^c^	0.8 ^c^	0.5 ^c^	1.3 ^c^	[48]

^a^: g/kg fresh weight, ^b^: g/100 g dry weight, ^c^: % Fatty acids. Column heading abbreviations are as follows: C10:0—capric, C12:0—lauric, C14:0—myristic, C15:0—pentadecanoic, C16:0—palmitic, C17:0—heptadecanoic, C18:0—stearic, C20:0—arachidic, C14:1—myristoleic, C16:1—palmitoleic, C17:1—heptadecenoic, C18:1—oleic, C20:1—eicosenoic, C18:2—linoleic, C18:3—linolenic, SFA—saturated fatty acids, MUFA—monounsaturated fatty acid, PUFA—polyunsaturated fatty acid. NA: not available.

**Table 4:** Vitamin composition and content of insects.

Scientific Name	A (IU/kg)	B_1_ (mg/kg)	B_2_ (mg/kg)	B_6_ (mg/kg)	B_12_ (μg/kg)	C (mg/kg)	E (IU/kg)	PP (mg/kg)	Reference
*Hermetia illucens*	<1000	7.7	16.2	6.01	55.8	<10.0	6.2	71.0	[49]
*Chilecomadia moorei*	<1000	<0.01	64.5	3.29	5.1	23.0	13.0	33.6	[49]
*Blatta lateralis*	<1000	0.9	15.6	3.10	237	<10.0	<3.3	43.8	[49]
*Musca domestica*	<1000	11.3	77.2	1.72	6.0	<10.0	29.7	90.5	[49]
*Zophobas morio*	<1000	0.6	7.5	3.2	NA	12.0	7.7	32.3	[33,50]
*Tenebrio molitor*	<1000	1.2	16.1	5.8	NA	24.0	<5.0	41.3	[33,50,51]
*Galleria mellonella*	<1000	2.3	7.3	1.3	NA	<10.0	13.3	37.5	[33,50]
*Bombyx mori*	1580	3.3	9.4	1.6	NA	<10.0	8.9	26.3	[33]
*Acheta domesticus*	<1000	0.4	34.1	2.3	NA	30.0	19.7	38.4	[33,50]
*Ruspolia differens*	<1000	NA	14	4.4	NA	6.2	22.6	36.1	[41,76]
*Macrotermes* *subhylanus*	<1000	NA	41.8	2.7	NA	7.3	NA	28.0	[76]
*Oecyphylla smaragdina*	NA	2.25	6.75	NA	NA	20.0	NA	NA	[39]
*Rhynchophorus ferrugi-neus*	NA	NA	NA	NA	NA	NA	18.8	NA	[48]

Column heading abbreviations are as follows: A—vitamin A, B_1_—thiamin, B_2_—riboflavin, B_6_—pyridoxine, B_12_—cobalamin, C—vitamin C, E—vitamin E, PP—niacin. NA: not available.

**Table 5:** Mineral composition and content of selected insects.

Scientific Name	Ca	P	Mg	Na	K	Cl	Fe	Zn	Cu	Mn	I	Se	Reference
*Apis mellifera*	849 ^a^	7825 ^a^	1770 ^a^	584 ^a^	18,719 ^a^	NA	133 ^a^	116 ^a^	36 ^a^	12 ^a^	NA	NA	[42]
*Gryllodes sigillatus*	1300 ^a^	NA	1010 ^a^	3330 ^a^	11,900 ^a^	NA	42.3 ^a^	139 ^a^	47.9 ^a^	NA	NA	NA	[45]
*Schistocerca gregaria*	700 ^a^	NA	820 ^a^	1730 ^a^	7490 ^a^	NA	83.8 ^a^	186 ^a^	63.2 ^a^	NA	NA	NA	[45]
*Ruspolia differens*	245 ^a^	NA	331 ^a^	1210 ^a^	2597 ^a^	NA	2297 ^a^	130 ^a^	25 ^a^	124 ^a^	NA	5 ^a^	[41,43]
*Gonimbrasia belina*	1278 ^a^	NA	697 ^a^	412 ^a^	102 ^a^	NA	267 ^a^	NA	3 ^a^	15 ^a^	NA	NA	[43]
*Gynanisa maja*	1664 ^a^	NA	1000 ^a^	324 ^a^	655 ^a^	NA	136 ^a^	NA	3 ^a^	14 ^a^	NA	NA	[43]
*Macrotermes falciger*	780 ^a^	NA	490 ^a^	127 ^a^	127 ^a^	NA	248 ^a^	NA	7 ^a^	15 ^a^	NA	NA	[43]
*Imbrasia belina*	570 ^a^	240 ^a^	NA	2670 ^a^	110 ^a^	NA	1160 ^a^	NA	NA	NA	NA	NA	[44]
*Gryllus bimaculatus*	2402 ^a^	11,696 ^a^	1467 ^a^	4530 ^a^	10,799 ^a^	NA	97 ^a^	224 ^a^	46 ^a^	104 ^a^	NA	NA	[37]
*Allomyrina dichotoma*	1234 ^a^	8607 ^a^	2836 ^a^	1484 ^a^	12,491 ^a^	NA	143 ^a^	103 ^a^	14 ^a^	86 ^a^	NA	NA	[37]
*Protaetia brevitarsis*	2587 ^a^	11,404 ^a^	3276 ^a^	2116 ^a^	20,014 ^a^	NA	162 ^a^	119 ^a^	18 ^a^	59 ^a^	NA	NA	[37]
*Teleogryllus emma*	1935 ^a^	10,854 ^a^	1525 ^a^	2782 ^a^	8955 ^a^	NA	108 ^a^	185 ^a^	22 ^a^	59 ^a^	NA	NA	[37]
*Rhynchophorus ferrugineus*	380 ^a^	2390 ^a^	1200 ^a^	380 ^a^	5680 ^a^	NA	10 ^a^	80 ^a^	11 ^a^	6 ^a^	NA	NA	[48]
*Hermetia illucens*	9340 ^b^	3560 ^b^	1740 ^b^	887 ^b^	4530 ^b^	1160 ^b^	66.60 ^b^	56.20 ^b^	4.03 ^b^	61.80 ^b^	0.26 ^b^	0.32 ^b^	[49]
*Chilecomadia moorei*	125 ^b^	2250 ^b^	278 ^b^	198 ^b^	2590 ^b^	1160 ^b^	14 ^b^	37.70 ^b^	2.95 ^b^	0.71 ^b^	0.10 ^b^	0.03 ^b^	[49]
*Blatta lateralis*	385 ^b^	1760 ^b^	250 ^b^	744 ^b^	2240 ^b^	1600 ^b^	14.80 ^b^	32.70 ^b^	7.93 ^b^	2.64 ^b^	0.30 ^b^	0.30 ^b^	[49]
*Musca domestica*	765 ^b^	3720 ^b^	806 ^b^	1380 ^b^	3030 ^b^	1760 ^b^	125 ^b^	85.80 ^b^	12.90 ^b^	26.60 ^b^	0.10 ^b^	0.15 ^b^	[49]
*Zophobas morio*	177 ^b^	2370 ^b^	498 ^b^	475 ^b^	3160 ^b^	1520 ^b^	16.5 ^b^	30.7 ^b^	3.6 ^b^	4.3 ^b^	<0.1 ^b^	0.14 ^b^	[33,50]
*Tenebrio molitor*	184 ^b^	2720 ^b^	864 ^b^	489 ^b^	2970 ^b^	1750 ^b^	21.5 ^b^	44.5 ^b^	6.4 ^b^	3.6 ^b^	<0.1 ^b^	0.13 ^b^	[33,50,51]
*Galleria mellonella*	234 ^b^	1950 ^b^	316 ^b^	165 ^b^	2210 ^b^	640 ^b^	20.9 ^b^	25.4 ^b^	3.8 ^b^	1.3 ^b^	<0.1 ^b^	0.11 ^b^	[33,50]
*Bombyx mori*	177 ^b^	2370 ^b^	498 ^b^	475 ^b^	3160 ^b^	620 ^b^	16.5 ^b^	30.7 ^b^	3.6 ^b^	4.3 ^b^	<0.1 ^b^	0.14 ^b^	[33]
*Acheta domesticus*	407 ^b^	2950 ^b^	337 ^b^	1340 ^b^	3470 ^b^	2270 ^b^	19.3 ^b^	67.1 ^b^	6.2 ^b^	11.5 ^b^	0.21 ^b^	0.19 ^b^	[33,50]

^a^: mg/kg dry weight; ^b^: mg/kg fresh weight. Column heading abbreviations are as follows: Ca—Calcium, P—phosphorus, Mg—magnesium, Na—sodium, K—potassium, Cl—chloride, Fe—iron, Zn—zinc, Cu—copper, Mn—manganese, I—iodine, Se—selenium. NA: not available.

**Table 6:** Biological functions of insect active ingredients.

Efficacy	Functional Ingredients	Origin (Latin Name of the Insect)	Cell Line/Animal Model	In Vivo/In Vitro	Effects	References
Anti-cancer	Amblyomin-X	*Amblyomma americanum*	Human melanoma (SK-Mel-28) and primary pancreatic adenocarcinoma (Mia-PaCa-2) cells	In vitro	Amblyomin-X targets the ubiquitin-proteasome system and cell cycle-related genes to promote tumor cell death.	[92]
	Bee venom	*——*	Human lung cancer cell lines A549 and NCI-H460	In vitro	By boosting the expression of death receptor 3 and deactivating NF-kappa β in non-small cell lung cancer cells, bee venom reduces the development of cancer cells.	[93]
	Glycosaminoglycan	*Catharsius molossus*	Melanoma mice induced by B16F10 cells	In vivo	By boosting TIMP-2 activity and adhesion activity, glycosaminoglycan can thicken the extracellular matrix, which in turn promotes the invasion and growth of tumor cells.	[94]
	72-kDa anticancer protein (EPS72)	*Eupolyphaga sinensis*	Human lung cancer A549 cell line	In Vitro	A549 cells are made to detach and undergo apoptosis when exposed to EPS72, which also prevents cell migration and invasion by impairing cell adherence to collagen IV and fibronectin.	[95]
	Se-rich amino acids	Ziyang Silkworm pupae	Human hepatoma cells	In Vitro	Se-rich amino acids can inhibit cell viability, induce changes in cell morphology and cycle, and induce apoptosis through the production of ROS.	[96]
	Protein	*Bombyx mori*	Human colon cancer cells DLD-1	In vitro	The protein from silkworm pupae prevents the growth of cancer cells, encourages apoptosis, and alters the energy metabolism of cancer cells by slowing down glycolysis and mitochondrial respiration.	[97]
	Protein hydrolysates	*Bombyx mori*	Human gastric cancer SGC-7901 cells	In vitro	Protein hydrolysates cause the accumulation of ROS, the depolarization of the mitochondrial membrane potential, and death in cancer cells while also blocking the S-phase cell cycle.	[98]
	Protein hydrolysates	*Bombyx mori*	MGC-803 gastric cancer cells	In vitro	Affects the metabolism of the MGC-803 cell energy supply.	[99]
	Ixolaris	*Ixodes scapularis*	U87-MG human glioblastoma cell lines	In vitro	The inhibitory effect of Ixolaris on tumor growth is associated with the downregulation of VEGF and reduced tumor angiogenesis.	[100]
	Protein extracts	*Bombyx mori*; *Samia ricini*	Breast cancer cells MCF-7	In vitro	The extracts dramatically decreased the levels of IL-6, IL-1, and TNF-α in MCF-7 cells as well as their protein and nucleic acid composition.	[101]
Antibacterial	Chitin film	*Blaberus giganteus*	*Aspergillus niger* (CBS 554.65)	In vitro	The hydrophobic properties of chitin film prevent microbial development.	[102]
	Bee venom and melittin	*——*	*Borrelia burgdorferi*	In vitro	All examined species of *Borrelia burgdorferi* were significantly affected by bee venom and melittin, which also suppressed the Lyme disease that *Borrelia burgdorferi* causes.	[103]
	Trx-stomoxynZH1	*Hermetia illucens*	*Bacterium*	In vitro	Trx-stomoxyn ZH1 exhibits different inhibitory activities against a variety of bacteria.	[104]
	Silk, Cecropin B	transgenic *Bombyx mori*	*E. coli* (ATCC 25922)	In vitro	It prevents Gram-negative *E. coli* from growing.	[105]
	Pygidial gland secretion	*Calosoma sycophanta*	*E. coli*	In vitro	When compared to effective medications, pygidial gland secretion exhibited stronger antifungal activity.	[106]
	Royalisin	*Apis mellifera*	*——*	In vitro	Royalisin has antibacterial activity against fungi and gram-positive and gram-negative bacteria.	[107]
	Chitin and chitosan	*Bombyx mori*	Bacillus cereus; Staphylococcus aureus; E. coli; Klebsiella pneumonia	In vitro	Compared to commercially available chitosan, it has stronger antibacterial properties.	[108]
	Pupal cell	*Curculio caryae*	Beauveria bassiana	In vitro	Entomopathogenic fungi are suppressed by pupal cells.	[109]
	Peptide fraction II	*Antheraea mylitta*	MDR Gram-negative bacteria	In vitro	The bacterial outer membrane may become pit-likely deformed as a result of peptide fraction II.	[110]
Antioxidant	Polypheno ls	*Bombyx mori*	——	In vitro	It has a strong capacity for scavenging ROS.	[111]
	water-soluble chitosan	*Clanis bilineata*	d-galactose-induced-aged mouse model	In vivo	Water-soluble chitosan dramatically boosted the activity of superoxide dismutase and glutathione peroxidase in mouse stomachs demonstrating strong scavenging ability against superoxide anions and hydroxyl radicals and prevented the generation of malondialdehyde in mouse brain and serum.	[112]
	extract oil	*Clanis bilineata*	——	In vitro	In experiments to prevent β-carotene from bleaching and scavenge DPPH radicals, extract oil demonstrated strong antioxidant activity.	[113]
	Sericin	*Antheraea mylitta*	——	In vitro	Researchers have discovered that sericin contains anti-tyrosinase, anti-elastase, glutathione-S-transferase activity inhibition, free radical scavenging potential, and inhibits lipid peroxidation properties.	[114]
	Protein hydrolysates	*Bombyx mori*	Hepatic HepG2 cells	In vitro	In HepG2 cells, protein hydrolysates had ROS reduction, SOD expression, and glutathione synthesis effects.	[115]
Hepatoprotection	Oil	*Bombyx mori*	Acetaminophen-induced acute liver injury Kunming mice model	In vivo	Silkworm oil reduces acute liver damage by blocking the NF-κB signaling pathway that is caused by oxidative stress.	[116]
Improves atherosclerosis	Crude extract	*Bombyx mori*	Male New Zealand white rabbits	in vivo	There are fewer atherosclerotic plaques in histopathology.	[117]
	Silkworm Protein 30Kc6	*Bombyx mori*	In vivo atherosclerosis rabbit model	in vivo	By lowering serum levels of total triglycerides (TGs), high-density lipoprotein cholesterol (HDL-C), low-density lipoprotein cholesterol (LDLC), and total cholesterol (TC), this protein reduces atherosclerosis in rabbits.	[118]
Antiviral	Alloferon	*Calliphora vicina*	the model of mice lethal pulmonary infection with human influenza viruses A and B	in vivo	Natural killer cells are stimulated by alloferon, which also causes the production of IFN in mice.	[119]
Anti-inflammatory	Glycosaminoglycan	*Gryllus* *bimaculatus*	Chronic arthritic rat model	in vivo	By inhibiting C-reactive protein (CRP) and rheumatoid factor, this GAG demonstrated a strong anti-edema impact. It also prevented atherogenesis by lowering proinflammatory cytokine levels.	[120]
	Protein-enriched fraction/extracts (PE)	*Musca domestica*	Male C57/BL6 inbred mice	In vivo	In experimental atherosclerotic lesions, PE is efficient at inhibiting a range of pro-inflammatory responses in vivo.	[121]
	Sialostatin L	*Ixodes scapularis*	Mouse cell line CTLL-2	In vitro	Sialostatin L possesses anti-inflammatory properties and prevents cytotoxic T lymphocyte proliferation.	[122]
	Non-peptide nitrogen compounds	*Polyrhachis dives*	Rat mesangial cells	In vitro	Non-peptide nitrogen compounds reduce inflammation by preventing the activity of COX-1, COX-2, and TNF-α.	[123]
	Venom	*Nasonia vitripennis*	Raw264.7 cells, murine fibrosarcoma L929sA cells, human embryonic kidney 293T cells	In vitro	In mammalian cells, it blocks the NF-κB signaling pathway.	[124]
	Peptides	*Gryllodes sigillatus, Tenebrio molitor, Schistocerca gragaria*	——	In vitro	The hydrolysates of edible insects include peptide fractions with significant lipoxygenase and cyclooxygenase-2 inhibitory activity.	[125]
Immunomodulatory activity	Bee Venom Phospholipase A2	Honeybee	BALB/c and C57BL/6 mice	In vivo	Bee Venom Phospholipase A2 Induces a Primary Type 2 Response that Is Dependent on the Receptor ST2 and Confers Protective Immunity	[126]
	Polysaccharide	*Bombyx mori*	Penaeid prawns	In vivo	Activated innate immunity in prawns.	[127]
	Peptides	*Bombyx mori*	Mouse spleen cells	In vitro	The expression of immune-related factors is stimulated by active peptides.	[128]
As medical biomaterial	Silk (cocoons)	*Hydropsyche* *angustipennis*	——	——	Silk is a promising biomaterial for tissue engineering since it may be utilized as a scaffold for cell growth.	[129]
Regulation of blood sugar and blood lipids	Glycosaminoglycan	*Gryllus bimaculatus*	Wistar rats	In vivo	Total cholesterol, phospholipid, and glucose levels decreased in the treated rats in a dose-dependent way, as did abdominal and epididymal fat.	[130]
	Protein hydrolysates	*Bombyx mori*	3T3-L1 cells	In vitro	Protein hydrolysates can boost leptin levels and boost GLUT4 levels to promote glucose uptake and decrease fat storage, respectively.	[131]
	Oil	*Bombyx mori*	Sprague– Dawley rats	In vivo	Silkworm pupa oil increases fat metabolism, which decreases blood lipid levels.	[132]
	Soluble fibroin	*Bombyx mori*	3T3-L1 adipocyte	In vitro	It improves glucose uptake and metabolism.	[133]
	Protein	*Bombyx mori*	Male C57BL/6mice	In vivo	Silkworm pupae protein dramatically lowers blood glucose levels in mice.	[134]
Blood pressure reduction	Peptide hydrolysates	Silkworm pupae	Spontaneously hypertensive rats	In vivo	In the treatment group, hypertensive mice’s systolic blood pressure dropped and was dose-dependent.	[135]
	Protein hydrolysates	*Bombyx mori*	RP- HPLC	In vitro	Silkworm protein hydrolysates have an inhibiting effect on the angiotensin I-converting enzyme.	[136]
Angiogenesis inhibition	Troponin I-like	*Haemaphysalis longicornis*	Human vascular endothelial cells	In vitro	Troponin I-like compounds effectively prevented human vascular endothelial cells from forming capillaries. There was evidence of a dose-dependent inhibition.	[137]
	Salivary gland extracts	*Ixodes scapularis*	microvascular endothelial cell	In vitro	Salivary gland extracts are negative regulators of angiogenesis-dependent wound healing and tissue repair and suppress the proliferation of microvascular endothelial cells in a dose-dependent way.	[138]
	Caffeic acid phenethyl ester	*Apis mellifera*	Human umbilical vein endothelial cells	In vitro	A potent inhibitor of vascular endothelial growth factor-induced angiogenesis is the caffeine acid phenethyl ester.	[139]
	Crude whole body extracts	*Tabanus bovinus*	Rat corneal model	In vivo	Extracts greatly decreased the length of blood vessels in the neovascularized cornea and the thick vascular networks emanating from the corneoscleral limbus.	[140]
	Haemangin	*Haemaphysalis longicornis*	Rabbits	In vivo	By preventing vascular endothelial cells from proliferating and triggering death, haemangin can impair angiogenesis and wound healing.	[141]
Anti-apoptotic	Silkworm Protein 30Kc6	*Bombyx mori*	The in vitro cell apoptosis model of HUVEC was induced by oxidized low-density lipoprotein.	In vitro	By obstructing the MAPK signaling pathway, 30Kc6 inhibits the death of HUVEC cells brought on by oxidized LDL.	[118]
	Storage protein 1	——	HeLa cells	In vitro	Storage protein 1 may operate as an upstream inhibitor of apoptosis since it decreases the loss of mitochondrial membrane potential and prevents caspase-3 activation.	[142]
	Recombinant 30 K protein	*Bombyx mori*	HeLa cells; Spodoptera Frugiperda (Sf9) cells	In vitro	In human and insect cells, the 30 K protein inhibits the apoptosis that is brought on by viruses or toxins.	[143]
Anti-genotoxic	Pupae extract	*Antheraea* *assamensis*	Normal human leukocytes	In vitro	DNA damage brought on by hydrogen peroxide was stopped by pupa extract at 1 mg/mL.	[144]
Wound healing	Honey	*Apis mellifera*	——	In vitro	A strong non-antibacterial chemical found in honey promotes the cells responsible for wound healing.	[145]
	Royal jelly protein 1	Honeybee	keratinocytes	In vitro	Keratinocytes are activated by royal jelly protein 1.	[146]
Anti-allergy	Royal jelly protein 3	Honeybee	OVA/alum-immunized mice	In vivo	Royal jelly protein 3 suppresses IL-4 production by activating splenocytes with anti-CD3 receptors.	[147]
Anti-fatigue agents	Silk powder	*Bombyx mori*	Imprinting Control Region (ICR) mice	In vivo	In mice, the silkworm powder was able to prolong swimming time and muscle mass while lowering exhaustion.	[148]
	Protein isolate	Buffalo larvae	Healthy young men	In vivo	Consumption of insect protein isolates improves individuals’ muscular strength.	[149]
Improves skin wrinkles	Honeybee-venom serum	*A. mellifera* L.	Healthy women	In vitro	By reducing the total wrinkle area, total wrinkle counts, and average wrinkle depth, bee venom serum can clinically improve facial wrinkles.	[150]
Regulation of intestinal flora	Whole worm	*Hermetia* *illucens*	Laying hens	In vivo	The species and relative abundance of gut bacteria are changed by consuming *Hermetia illucens*.	[151]
	Partially defatted meal of larvae	Rainbow trout	Rainbow trout	In vivo	The diversity of the gut flora has risen, and its community organization has changed.	[152]
	Whole cricket powder	Cricket	Healthy adults	In vivo	Consuming cricket powder can help probiotics grow and minimize inflammatory responses.	[153]
Anti-HIV	Venom peptide	Honeybees	HIV cells	In vitro	HIV-infected cells that absorb bee venom peptides exhibit decreased HIV gene expression and replication.	[154]
Treatment of Parkinson’s disease	Bee Venom	Honeybee	Chronic mouse model of MPTP/probenecid	In vivo	In an animal model modeling the chronic degenerative process of Parkinson’s disease, bee venom offers long-lasting protection.	[155]
Anti-Alzheimer’s disease	Silkworm pupae Powder	*Bombyx mori*	Male Wistar rats	In vivo	In vivo, hippocampus memory impairments and hippocampal neuron density in mice were both considerably enhanced by silkworm pupae powder.	[156]
	Silkworm pupa vaccine	*Bombyx mori*	Transgenic mouse model of AD	In vivo	In AD mice, it enhances memory and cognitive function.	[157]
Alcohol detoxification	Extracts	*Bombyx mori*	ICR mice	In vivo	Alcohol dehydrogenase activity in the liver was dramatically boosted by oral administration of silkworm pupa extract at 0.5 mg/mL.	[158]
Treatment of gastric ulcers	Oil	*Bombyx mori*	Hydrochloric acid/ethanol-induced gastric ulcers Kunming mice model	In vivo	Gastric ulcers can be treated with silkworm pupa oil by shrinking the ulcer and lessening the inflammatory response.	[159]
Promotes hair growth	Fermented cricket powder	*Gryllus bimaculatus*	Male C57BL/6 mice	In vivo	By controlling the expression of growth factors, the amino acids and other trace components in fermented cricket powder enhance hair development.	[160]

“——” indicates not stated in the literature.

## Data Availability

Not applicable.
